# Integrated transcriptomics and miRNAomics provide insights into the complex multi-tiered regulatory networks associated with coleoptile senescence in rice

**DOI:** 10.3389/fpls.2022.985402

**Published:** 2022-10-12

**Authors:** Jyothish Madambikattil Sasi, Cheeni VijayaKumar, Bharti Kukreja, Roli Budhwar, Rohit Nandan Shukla, Manu Agarwal, Surekha Katiyar-Agarwal

**Affiliations:** ^1^ Department of Plant Molecular Biology, University of Delhi South Campus, New Delhi, India; ^2^ Department of Botany, University of Delhi, Delhi, India; ^3^ Bionivid Technology Pvt. Limited, Bengaluru, Karnataka, India

**Keywords:** rice, coleoptile, senescence, transcriptome, miRNAs, regulation

## Abstract

Coleoptile is the small conical, short-lived, sheath-like organ that safeguards the first leaf and shoot apex in cereals. It is also the first leaf-like organ to senesce that provides nutrition to the developing shoot and is, therefore, believed to play a crucial role in seedling establishment in rice and other grasses. Though histochemical studies have helped in understanding the pattern of cell death in senescing rice coleoptiles, genome-wide expression changes during coleoptile senescence have not yet been explored. With an aim to investigate the gene regulation underlying the coleoptile senescence (CS), we performed a combinatorial whole genome expression analysis by sequencing transcriptome and miRNAome of senescing coleoptiles. Transcriptome analysis revealed extensive reprogramming of 3439 genes belonging to several categories, the most prominent of which encoded for transporters, transcription factors (TFs), signaling components, cell wall organization enzymes, redox homeostasis, stress response and hormone metabolism. Small RNA sequencing identified 41 known and 21 novel miRNAs that were differentially expressed during CS. Comparison of gene expression and miRNA profiles generated for CS with publicly available leaf senescence (LS) datasets revealed that the two aging programs are remarkably distinct at molecular level in rice. Integration of expression data of transcriptome and miRNAome identified high confidence 140 miRNA-mRNA pairs forming 42 modules, thereby demonstrating multi-tiered regulation of CS. The present study has generated a comprehensive resource of the molecular networks that enrich our understanding of the fundamental pathways regulating coleoptile senescence in rice.

## Introduction

Plant senescence, the terminal phase of plant development, is a regimented degradation process which ensures efficient nutrient recycling, fitness and reproductive success. It is a natural phenomenon primarily governed by developmental age and has a multi-layered regulation in which phytohormones play a pivotal role in integrating developmental signals with extrinsic factors, thereby fine-tuning the senescence program by an intricate network of signaling pathways (Lim et al., 2007). Abscisic acid (ABA), jasmonic acid (JA), salicylic acid (SA), ethylene, brassinosteroids (BRs) and strigolactones are well-acknowledged positive regulators of plant senescence, while cytokinins, gibberellins and auxins are generally known to negatively regulate plant senescence. Eventually all the cells and tissues undergo slow death in the plant organs that are destined for senescence. Among different plant organs, extensive studies have been carried out for understanding the molecular mechanism underlying the regulation of leaf senescence in plants. The senescence-associated deterioration process is tightly controlled at transcriptional, post-transcriptional and epigenetic level. The transcriptional regulation of senescence refers to the changes in the expression pattern of genes which are known as senescence-associated genes (SAGs). Several transcriptome studies have revealed massive reprogramming of a large repertoire of genes predominantly belonging to families such as transcription factors (TFs), proteases, receptor-like kinases, and nucleases. Members of TF families such as NAC, WRKY, bHLH, Zinc finger, MYB, TCP and AP2 participate in the senescence programme and many of these represent nodal points for mediating crosstalk between external and internal factors to initiate the aging process. Like many other processes of plant development, the last stage is also regulated at post-transcriptional level by small non-coding RNAs (Thatcher et al., 2015). High-throughput sequencing has fuelled the discovery of a large number of miRNAs associated with leaf senescence in multiple plant species (Xu et al., 2014; Huo et al., 2015; Wu et al., 2016). Three miRNA regulatory modules and their cognate TF targets i.e., miR164-ORE1, miR319-TCP4 and miR390-TAS3-ARF2 play critical role in regulating leaf senescence in Arabidopsis (Ellis et al., 2005; Kim et al., 2009; Li et al., 2013). Senescence is also regulated epigenetically by DNA methylation, histone modifications and chromatin alterations (Ay et al., 2014). Overall, the complex regulatory interactive network comprising SAGs (mainly TFs), miRNAs, hormones and chromatin modifications modulate leaf senescence in plants.

Among different plant organs, coleoptile is the first organ to undergo senescence post-germination in cereal plants. It is a protective sheath that emerges two days after sowing and covers emerging shoots in monocotyledonous plants. In addition to providing physical protection to first true leaves, the coleoptile also provides nutrition, mainly carbohydrates, to the growing parts of developing seedlings (Fröhlich and Kutschera, 1995). During seed germination under submerged conditions, coleoptile rapidly elongates and acts like a ‘snorkel’ to uptake oxygen from the water surface and transfer it to the underwater parts for their optimal growth (Narsai et al., 2015). It is evidently clear that the coleoptile, along with the first leaf, play a crucial role in seedling establishment, which is a key determinant of plant fitness and productivity of the cereal crops. Interestingly, the coleoptile continues to elongate under anaerobic conditions, however, on transfer to aerobic conditions it rapidly attains maturation followed by growth cessation and initiation of senescence (Inada et al., 2002). Under optimum growth condition, the coleoptile turns green from white within 3 days of seedling transfer to air. This is followed by emergence of the first leaf along with splitting of coleoptile in the next 24 h. Senescence of coleoptile is initiated rapidly afterwards and is evident by the formation of lysigenous aerenchyma, chlorophyll degradation and DNA fragmentation (Inada et al., 2002). Other than coleoptile, leaf senescence also occurs naturally in rice plants. Though deterioration events such as chloroplast breakdown followed by nuclear condensation are conserved in coleoptile and leaf senescence, aging within an individual leaf is highly asynchronous, thereby making it difficult to accurately characterize its senescence program and associated regulatory pathways (Inada et al., 1998). On the contrary, the senescence in coleoptile is relatively synchronous because of its simple cellular architecture consisting of only two vascular bundles and few layers of mesophyll cells. Owing to the simple cellular architecture, short life-span and quick and uniform pattern of aging, coleoptile is often proposed as a model organ for studying the mechanism of senescence in cereals.

Comprehensive understanding of the senescence process in coleoptile has been gained with respect to the pattern of cell death events and major changes that occur at tissue and cellular level, however, till date there is no genomic-scale study on discovering the gene expression changes during coleoptile senescence in any cereal plant. With an aim to gain insights into the components of regulatory pathway(s) and possible interaction among these components/pathways that contribute to coleoptile senescence, we employed an integrated omics approach and performed high-throughput sequencing of coding as well as non-coding RNAs to identify the differentially expressed genes and their regulation by miRNAs. A comparative analysis of the coleoptile transcriptome dataset with the publicly available leaf senescence dataset revealed the unique and common components between the two senescing organs. Efforts were made to validate some of the transcripts and miRNAs that exhibited differential expression during senescence. Regulatory networks involving differentially expressed genes and their probable miRNA regulators were integrated with protein-protein interaction (PPI) datasets, which vividly supported a multi-tiered regulation of senescence program in rice coleoptiles.

## Materials and methods

### Plant material

The surface-sterilized rice seeds (var. Pusa Basmati 1) were submerged in autoclaved water for seven days at 28^0^C in test tubes (8-10 cm below the water surface) placed in a growth room. After seven days of growth under submergence, the seeds with elongated coleoptiles were transferred to cotton beds soaked with rice growth media (Yoshida et al., 1976). Immediately after transferring to aerobic conditions, the unsenesced elongated coleoptile tissues (control-Day 0) were harvested. Senescence of coleoptiles was monitored on Day 1 and Day 2 under aerobic conditions, and tissues were harvested at each time point. Tissues from three biological replicates were harvested, frozen in liquid nitrogen and stored in -80°C for future use. Out of the three replicates, two replicates of control and Day 2 tissues were employed for high-throughput sequencing of RNA and small RNA. For quantitative PCR experiments three biological replicates comprising all three timepoints (control, Day 1 and Day 2) were utilized.

### mRNA and small RNA library preparation and high-throughput sequencing

Total RNA was isolated from the harvested tissues using a modified GITC method (Chomczynski and Sacchi, 1987). The quantity and quality of the isolated RNA were evaluated using a spectrophotometer (Bio-Rad, USA) and MOPS-formaldehyde-agarose denaturing gel electrophoresis. Four RNA-Seq libraries were constructed from control (Control_BR1 and Control_BR2) and Day 2 (Day 2_BR1 and Day 2_BR2) samples followed by their 101 bp paired-end sequencing using Illumina’s HiSeq4000 sequencing platform (Illumina, Inc., USA). For mRNA library preparation, RNA integrity of the control and Day 2 samples were assessed using Agilent Bioanalyzer 2100 system (Agilent Technologies, USA). TruSeq RNA Sample Preparation Kit v2 (Illumina, Inc., USA) was used according to the manufacturer’s instructions to construct the RNA sequencing libraries. The transcriptome library preparation and sequencing were outsourced to M/s Bionivid Technology Pvt. Ltd., India.

For small RNA library preparation, total RNA was resolved on TBE-urea-PAGE gel and the 15-30 nt portion corresponding to the low molecular weight (LMW) RNA fraction was excised and extracted for LMW RNA. The extracted LMW RNA was ligated with RNA adapters (5’ and 3’) using T4 RNA ligase and subsequently used for first-strand cDNA synthesis using Superscript III reverse transcriptase (Invitrogen, USA). A 3’ adapter specific reverse primer was used for the first-strand cDNA synthesis (**Supplementary Table 11**). The cDNA library was enriched and purified before quality check using Bioanalyzer (Agilent Technologies, USA). The small RNA-cDNA libraries were sequenced using Illumina HiSeq4000 sequencing platform by M/s Bionivid Technology Pvt. Ltd., India. The transcriptome and small RNA sequencing datasets were submitted to the SRA database as accession nos. GSE199139 and GSE199238, respectively.

### Differential gene expression analysis, gene ontology and functional enrichment analysis

The sequencing data obtained by RNA sequencing was analyzed for quality assessment. In brief, filtering of high quality (HQ) reads was performed with ‘NGS QC Toolkit (Patel and Jain, 2012). The HQ reads with an average Phred score ≥20 and ≥70% high-quality bases were included in the downstream analysis. *Oryza sativa*-Nipponbare-Reference-IRGSP-1.0 was used as the reference genome for mapping HQ adapter-trimmed reads. TopHat v2.0.11 was employed for mapping with default parameters (Trapnell et al., 2009). Subsequently, Cufflink (Cufflink_2.2.1) and Cuffmerge (Trapnell et al., 2009) tools were used for reference-based assembly of the reads. Fragments per transcript kilobase per million fragments mapped (FPKM) for each transcript was calculated from the number of mapped reads of the transcripts. Transcripts with normalized FPKM value ≥1 were considered for differential expression analysis with the Cuffdiff module of Cufflink. A comparative analysis of FPKM values of all the transcripts from Day 2 with that of control tissue was performed for identifying differentially expressed genes (DEGs). Identification of DEGs in coleoptile senescence was performed by calculating log (base 2) of FPKM of senescence sample divided by FPKM of control condition sample. A fold change of ±2 in the log2 scale was set as a cutoff for the categorization of DEGs. For the transcripts which were either specifically expressed in control or Day 2, fold change could not be calculated owing to FPKM value 0 in one of the samples. Since there were two biological replicates p-value was calculated to identify the transcripts which exhibited significant differential expression during senescence in the two biological replicates and transcripts with p-value <0.05 were shortlisted as a sub-dataset for downstream analyses.

GO ontologies were carried out using DAVID Functional Annotation Tool (DAVID Bioinformatics Resources 6.8, NIAID/NIH), and functional enrichment analysis was performed using MapMan annotation tool (http://mapman.gabipd.org/).

### Small RNA analysis and target prediction

The UEA Small RNA Workbench (version 2.4) was used to analyze purity-filtered raw reads (Stocks et al., 2012). The adapter sequences were removed from the raw reads and the resulting reads were converted into FASTA format by using the Adapter removal tool. Further, the reads were filtered according to the length and complexity. The invalid sequences, tRNAs, rRNAs and other non-coding RNA sequences were removed by filtering tools. For predicting mature miRNAs (novel and conserved), the miRCat tool was employed. Further, the Reference genome (*Oryza sativa* -Nipponbare-Reference-IRGSP-1.0) was used for mapping. miRBase v21 was used to identify the conserved miRNAs wherein two mismatches in the miRNA sequence were allowed. After filtering conserved miRNAs, novel miRNA predictions were carried out in the remaining small RNA population. Furthermore, the putative miRNAs were also screened manually according to criteria proposed by Meyers et al. (2008) and Kozomara and Griffiths-Jones (2014). To identify differentially expressed miRNAs, read counts were normalized and fold change was calculated as described by Xin et al. (2010). For target prediction, Plant small RNA target server (psRNATarget; https://www.zhaolab.org/psRNATarget/) was employed with default parameters and the expectation value was set to 4. MSU rice genome annotation, version 7 was used as the reference genome dataset. To generate a regulatory network of miRNA and target genes, we identified the predicted target IDs in the coleoptile transcriptome dataset generated using the VLOOKUP function in Microsoft excel.

### Gene expression profiling and validation of miRNAs

For the validation of transcriptome data, SYBR green based-qPCR method was employed, and the rice *eEF1α* was employed as an internal control for normalization. For validation and evaluation of expression of predicted rice miRNAs, poly A tailing method of qPCR method was employed with SYBR chemistry. 2 µg total RNA was poly-adenylated by using poly A polymerase enzyme (Ambion, USA) followed by a purification step using the RNAeasy MinElute Cleanup Kit (Qiagen, GmBH). The RTQ primer (**Supplementary Table 11**) along with Superscript III reverse transcriptase (Invitrogen, USA) was used to reverse transcribe poly A-tailed RNA population. The universal reverse primer and miRNA-specific forward primer were used for Real-time PCR amplification in Mastercycler Realplex 2 (Eppendorf, Germany) with KAPA PROBE FAST Universal qPCR kit (KAPA Biosystems, USA). For the normalization, rice 5S ribosomal RNA was used as an endogenous control. Relative expression levels were calculated after normalization against the control sample. Fold change was calculated using 2^-ΔΔct^ method (Livak and Schmittgen, 2001) and an average of all the replicates was plotted along with the calculated standard error (SE).

### Protein-protein interaction network creation and visualization

The protein-protein interaction of the high confidence putative miRNA target genes which exhibited inverse expression patterns in the transcriptome data with respect to the corresponding miRNA profile were analyzed using the STRING 11 database. The output obtained was integrated with the miRNAs and their predicted targets to generate the miRNA-target-PPI network using Cytoscape_v3.7.2 software.

## Results

### Characterization of coleoptile senescence at morphological and molecular level

**Figure 1:**
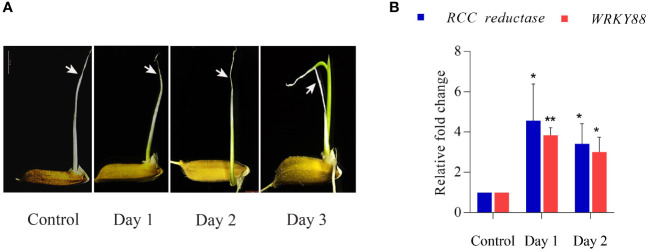
Coleoptile senescence in rice. **(A)** Different stages of coleoptile senescence in rice seedlings after transfer from anaerobic (submergence) to aerobic conditions. **(B)** Expression profile of senescence associated genes (*SAGs)* at different stages of coleoptile senescence using quantitative PCR. For normalization *eEF-1α* was used as an internal control. Three biological replicates and two technical replicates were included in the study. White arrows highlight the progression of senescence in the coleoptile. Asterisks indicate a significant difference between the control and senescence tissues (Student’s *t*-test, *P < 0.05, **P < 0.01). *RCC reductase: red chlorophyll catabolite reductase*.

With an aim to obtain elongated coleoptiles with synchronized growth and senescence, rice seeds were grown under submerged conditions for seven days. To induce rapid maturation and initiate senescence in coleoptiles, submerged seedlings were transferred to aerobic conditions (taken as ‘control’), and progression of senescence was subsequently monitored on day 1, day 2 and day 3 as depicted in **Figure 1A**. Closer examination of morphological alterations during coleoptile senescence revealed that wilting, curling and yellowing/browning of the coleoptile, especially at tips, was discernible at day 2 stage. On day 3, while the first leaf emerged, there was extensive wilting and browning of coleoptiles indicating late stage of senescence. Physio-morphological changes during progression of coleoptile senescence were confirmed at the molecular level by determining relative gene expression levels of senescence-associated marker genes (SAGs): *RED CHLOROPHYLL CATABOLITE REDUCTASE (RCC reductase)* and *WRKY88*. Increased expression of *RCC reductase* gene encoding for RCC reductase enzyme (which catabolizes chlorophyll) is a hallmark of senescence in leaves and other plant organs (Tang et al., 2011). Similarly, increased expression of *WRKY88* is reported during flag leaf senescence in rice (Lee et al., 2017). Quantitative PCR analysis demonstrated significant upregulation in steady state mRNA levels of both the genes during the progression of coleoptile senescence. The level of expression dramatically increased on day 1 and day 2. Notably, *RCC reductase* exhibited higher levels of induction at all the stages as compared to that of *WRKY88* (**Figure 1B**). These findings ratify the staging of coleoptile senescence in the present study. In view of these observations, day 2 was considered as the middle stage of coleoptile senescence which could be utilized for studying the changes occurring at molecular level by transcriptome and small RNAome analysis.

### Transcriptome sequencing, differential gene expression analysis and GO enrichment of differentially expressed genes during coleoptile senescence

**Figure 2:**
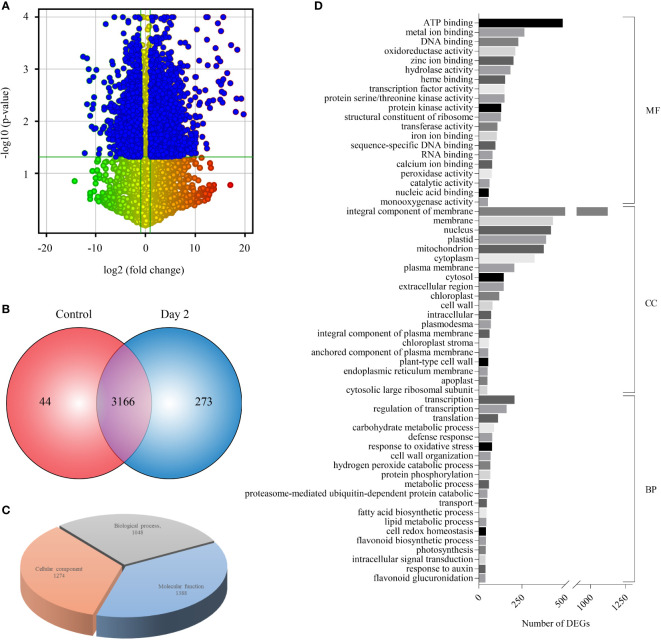
Expression profiling and GO enrichment of differentially expressed genes (DEGs) during coleoptile senescence in rice. **(A)** Volcano scatter plot depicting the expression pattern of DEGs in RNA-Seq data of rice coleoptile senescence. Statistically significant downregulated (top left) and upregulated (top right) genes in day 2 of senescence relative to control coleoptiles are highlighted in blue color. **(B)** Venn diagram representing the number of DEGs specific to control and senesced coleoptiles, and the ones which were expressed commonly in the two samples. **(C)** Gene Ontology (GO) enrichment analysis of DEGs. **(D)** Top twenty GO enrichment terms belonging to 3 separate sub-ontologies: molecular function (MF), cellular component (CC), and biological process (BP) and number of DEGs classified in each term are presented.

Sequencing of four RNA-Seq libraries [control (Control_BR1 and Control_BR2) and Day 2 (Day 2_BR1 and Day 2_BR2)] yielded an aggregate of 119.82 million HQ reads which were processed to calculate normalized FPKM values (**Supplementary Table 1**). Scatter plots plotted for comparing gene expression between the two independent biological replicates of control and day 2 samples showed good correlation (**Supplementary Figures 1A, B**). As evident from the volcano plot, the number of significantly upregulated transcripts (1970) were higher compared to the downregulated ones (1195) (**Figures 2A, B**). Overall, 3166 transcripts were differentially expressed (DEGs) in senesced coleoptile samples when compared to the control, while 44 and 273 genes exhibited specific expression in control and senesced coleoptiles, respectively (**Figure 2B**; **Supplementary Table 3**). These observations indicate that a large number of genes are regulated, during progression of senescence in rice coleoptiles. The genes exclusively regulated during senescence at Day 2 were also included under DEGs for further downstream analyses. To predict the function of genes exhibiting altered expression, GO analysis was carried out and GO terms could be assigned to 1792 out of 3439 DEGs. Maximum number of genes were associated with molecular function (77%) followed by cellular components (71%) and biological processes (58%) (**Figure 2C**, **Supplementary Table 3**). The top terms enriched under molecular function, cellular component and biological process GO categories were “integral component of ATP binding”, “integral component of membrane”, and “transcription” respectively (**Figure 2D**).

### Functional category enrichment analysis of differentially expressed genes during coleoptile senescence

**Figure 3:**
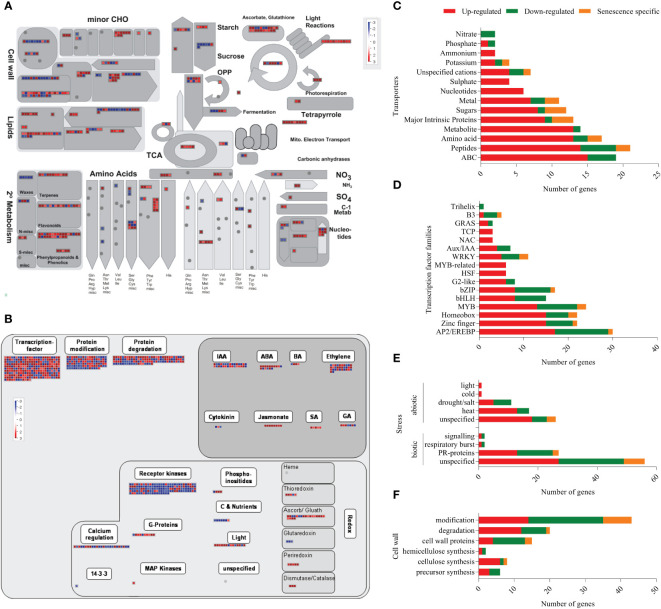
Functional annotation of DEGs using MapMan tool. The functional annotation of DEGs were analysed using MapMan tool and overviews of metabolism **(A)** and regulation **(B)** are depicted. In the overviews, red boxes represent up-regulated/senescence specific transcripts and the blue boxes represent down-regulated transcripts. The major functional categories of genes: Transporters **(C)**, Transcription factors **(D)**, Stress **(E)** and Cell wall-related **(F)** are represented as stacked bar graphs. In the stacked bar graphs red bar indicates upregulated genes, green bar shows down regulated genes and orange bar represents senescence-specific genes (day 2-specific).

To predict the functional categories associated with the DEGs, enrichment analysis was performed using the MapMan annotation tool (Thimm et al., 2004). Out of the 3439 DEGs, 438 and 823 genes mapped to the metabolism and regulation pathways, respectively (**Figures 3A, B**). Genes belonging to different functional categories such as transcriptional regulators (RNA category), transporters, hormone metabolism, redox related, stress response and signalling pathways are already known to play a role in the senescence of leaves (Guo et al., 2004; Gregersen and Holm, 2007; Zhang et al., 2014; Lin et al., 2015) and our analyses showed that 176, 37, 183, 134, 143, 94 and 105 transcripts belonging to signaling, redox, transcription factors, transporters, stress, cell wall and hormone signalling were differentially expressed during coleoptile senescence, respectively (**Figure 3**; **Supplementary Figure 2**; **Supplementary Table 4**). Detailed analysis of the selected categories revealed that out of the 176 signaling-related genes only 3 genes were specifically expressed in senescing coleoptiles. A greater number of signaling genes displayed downregulation (108) as compared to those that exhibited upregulation (65) (**Supplementary Figure 2A**; **Supplementary Table 4**). More genes belonging to receptor kinases (81), calcium (15) and sugar and nutrient (6) categories were downregulated; whereas higher numbers of genes from G-proteins (7) and light (11) categories exhibited upregulation. Relatively fewer genes belonging to receptor kinases (2) and G-proteins (1) were specifically expressed in senescing coleoptiles (**Supplementary Figure 2A**; **Supplementary Table 4**). Of the 37 redox-related transcripts, 29 and 8 were up- and down-regulated, respectively. While the entire set of DEGs grouped under sub-categories ‘dismutases, catalases, peroxiredoxin’ (17) were exclusively expressed in senescing tissue, all the 3 genes belonging to glutaredoxins category were exclusively expressed in control coleoptiles. A larger number of genes belonging to ‘ascorbate and glutathione’ (17) and thioredoxin (4) were upregulated (**Supplementary Figure 2B**; **Supplementary Table 4**). Of the 134 transporters identified, 98 were upregulated, 22 were downregulated and 14 were specifically expressed during coleoptile senescence. The transporter classes whose gene expression was upregulated majorly belonged to ABC (15), peptide (14), amino acid (13), metabolite (13), major intrinsic proteins or MIPs (11), sugar (8) nucleotide (6), ammonium (3), potassium (3) and sulphate (3) transporters. Whereas, genes of ABC (4), peptide (5), amino acid (2), metal (2) and nitrate (2) class of transporters were also found to be downregulated (**Figure 3C**; **Supplementary Table 4**). In the transcription factor category, 112, 61 and 12 genes belonging to 16, 12 and 8 TF families exhibited upregulation, downregulation and specific expression during coleoptile senescence, respectively. Maximum number of upregulated genes were of AP2/EREBP (17) TF family, followed by Zinc finger (15), Homeobox (15), MYB (13), bHLH (8), bZIP (8), G2-like (6), HSF (6), MYB-related (6) and WRKY (5). Furthermore, the main TF families whose transcripts showed downregulation were Zinc finger (6), AP2/EREBP (12), Homeobox (5), MYB (9), bHLH (7), bZIP (8), G2-like (2), WRKY (4), Aux/IAA (3) and B3 (3). Senescence-specific expression was observed for only a few transcripts, which were members of the zinc finger (1), AP2/EREBP (1), Homeobox (4), MYB (2), bZIP (1), WRKY (2) and B3 (1) TF families (**Figure 3D**; **Supplementary Table 4**).

Out of the 143 stress-associated genes, 56 belonged to abiotic stresses category (37 upregulated, 15 downregulated and 4 senescence-specific), while 87 were related to biotic stresses (44 upregulated, 34 downregulated and 9 senescence-specific) (**Figure 3E**; **Supplementary Table 4**). One of the limitations of the MapMan tool is that many annotated genes are not associated with any specific pathway and are listed as ‘unspecified’. Consequently, 56 and 19 genes under biotic and abiotic stress categories, respectively, were not assigned any sub-category. However, from the ones that were assigned a sub-category, PR proteins (13 upregulated, 12 downregulated and 3 senescence-specific) constituted a large number of differentially expressed biotic stress genes. In case of abiotic stresses, many heat stress (13 upregulated, 4 downregulated) and drought/salt stress (5 upregulated, 6 downregulated) related transcripts showed altered expression during coleoptile senescence. Because of extensive changes that occur in the cell wall during senescence (Serafini-Fracassini et al., 2002), we also analyzed genes associated with the term ‘cell wall’ and found that 94 genes associated with the cell wall exhibited differential expression. These included 42 downregulated, 40 upregulated, and 12 senescence-specific genes. Overall, genes associated with the degradation and cellulose synthesis were largely up-regulated, whereas genes associated with modification and cell wall proteins were down-regulated during senescence (**Figure 3F**; **Supplementary Table 4**).

Phytohormones regulate multiple aspects of plant growth and development (Gray, 2004). Therefore, we surveyed 105 genes belonging to the hormone signalling category and found that the number of upregulated transcripts (61) was higher than that of the downregulated (38) ones. In addition, few members (6) belonging to brassinosteroid (1), ethylene (2), SA (2) and cytokinin (1) signaling were exclusively expressed in the senescing tissue. The upregulated hormone signalling pathways include ethylene (17), auxin (15), ABA (12), JA (8), SA (3), gibberellic acid (4), and brassinosteroid (2). It is worthwhile to highlight that all the identified genes of JA and SA pathways exhibited dramatic upregulation. Whereas down-regulation of several transcripts belonging to auxin (15), ethylene (13), gibberellic acid (3), cytokinin (2), ABA (4) and brassinosteroid (1) was noted in coleoptile senescence (**Figure 3B**; **Supplementary Figure 2**; **Supplementary Table 4**).

### Comparison of transcriptome datasets of coleoptile and leaf senescence in rice

**Figure 4:**
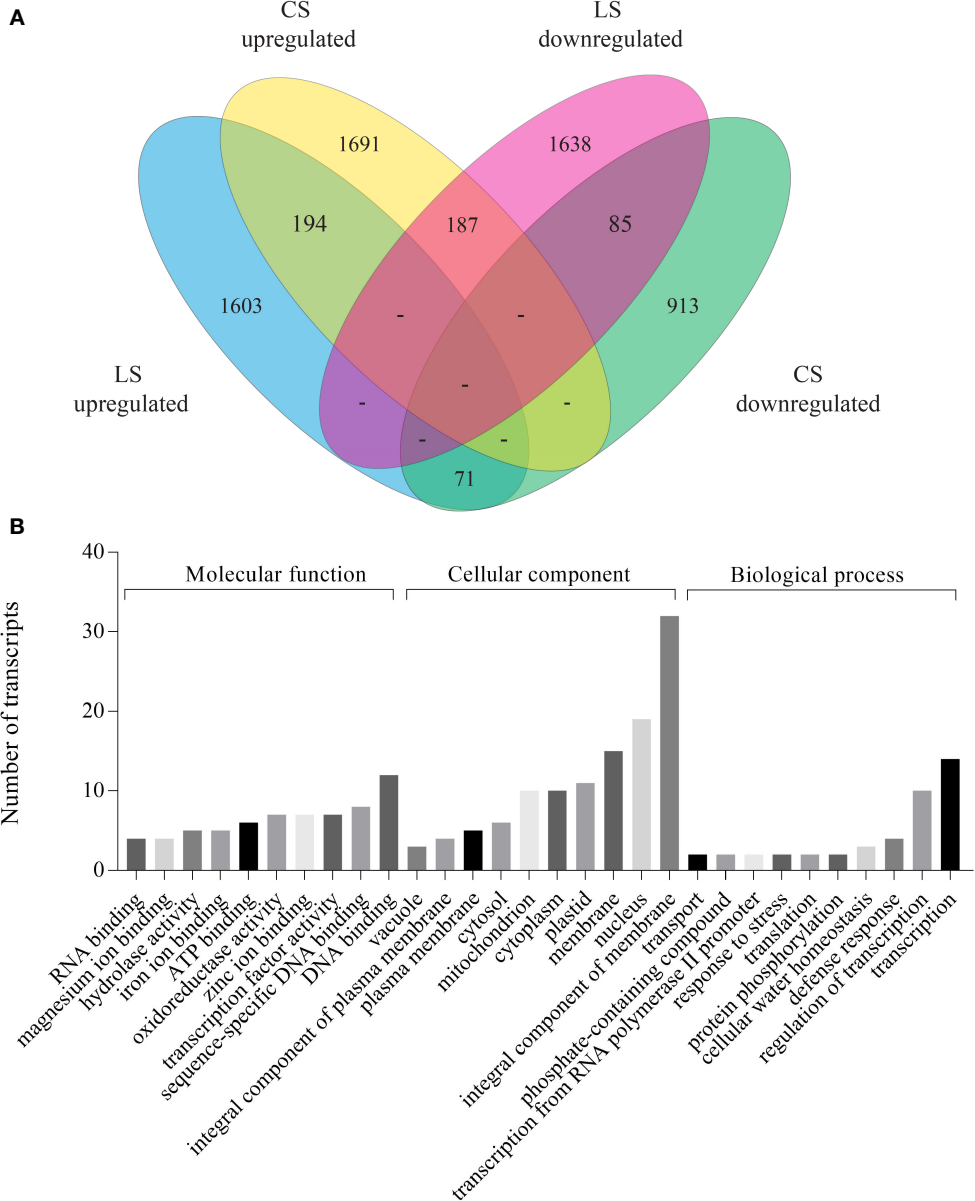
Comparison of DEGs identified in coleoptile senescence with those reported in natural leaf senescence in rice. Leaf senescence data (both second and flag leaf) was retrieved  (Lee et al., 2017) and was compared with coleoptile senescence dataset obtained in the present study. DEGs identified in the 36 days post heading (36 DPH) tissues of LS dataset were used for the analysis. **(A)** Venn diagram of DEGs shows overlap between CS and LS. **(B)** GO enrichment analysis of DEGs which were commonly upregulated during leaf as well as coleoptile senescence in rice.

To delineate the core and specific components involved in the two physiologically different senescence programs in plants, we compared the DEGs of coleoptile transcriptome (3439 DEGs) with the publicly available combined transcriptome data of second and flag leaf senescence, henceforth referred to as LS (3778 DEGs; Lee et al., 2017). While the majority of genes were specifically up- and down-regulated in LS and CS (1603, 1691 up-regulated and 1638, 913 down-regulated in LS, CS, respectively), a smaller, albeit significant, number of genes exhibited similar expression pattern in both LS and CS (194 showed upregulation and 85 were downregulated). These genes probably constitute the core components of senescence in rice. Based on these observations it is evident that CS is remarkably distinct from the LS program in rice. This is further supported by an inverse expression pattern of a substantial number of genes (258) in LS and CS (**Figure 4A**; **Supplementary Table 5**). To obtain insights into the biological function of core components of the senescence process, GO analysis of the commonly upregulated genes was carried out. The analysis revealed that maximum number of upregulated genes were associated with cellular components (enrichment of subcategories such as “integral components of membrane”, “nucleus” and “membrane”) followed by molecular function (subcategories such as “DNA binding” and “TF activity”) (**Figure 4B**; **Supplementary Table 5**). Functional enrichment analysis identified several transporters (amino acid transporters, aquaporins, MATE efflux protein, ion transporters), TFs (MYB, NAC, homeobox domain, Zinc finger proteins, and WRKY family proteins), cytochrome P450, heat shock transcription factors or HSFs (HSFA7a, HSFA3a and HSFC1b), hormone signaling pathway genes (ABA, auxin, ethylene and gibberellin) and chlorophyll degradation genes (RCC reductase and senescence-inducible chloroplastic stay green 1 protein) among the commonly upregulated genes (**Supplementary Table 5**). Genes encoding for 1-aminocyclopropane-1-carboxylate oxidase proteins, TFs such as MYB, bZIP, WRKY118, FBXs (FBX27, FBX 28), receptor like protein kinases, lectin-like receptor kinases and certain enzymes such as GDSL-like lipase/acid hydrolase and glycosyl hydrolase showed down regulation in both CS and LS. Among the genes that exhibited inverse correlation in expression during LS and CS, a large number of genes encoded for proteins belonging to subcategory “integral component of membrane” followed by ‘plastid’. Besides, during CS several genes associated with “secondary metabolite synthesis”, “photosynthesis”, “metal ion binding”, “oxidoreductases” and “response to stress” categories were significantly upregulated whereas they exhibited downward trend in expression in LS. On the other hand, several genes coding for proteins of subcategories “plasma membrane”, “DNA binding” and “transcription” showed significant induction in LS while their expression declined in CS (**Supplementary Table 5**). It is noteworthy that several membrane proteins, especially transporters (amino acid transporter, aquaporin, malic acid transporter, phosphate transporter and potassium transporter), were commonly upregulated, while some membrane proteins (citrate transporter, amino acid transporter) exhibited inverse correlation in their expression in CS and LS.

### Validation of rice coleoptile senescence transcriptome data

**Figure 5:**
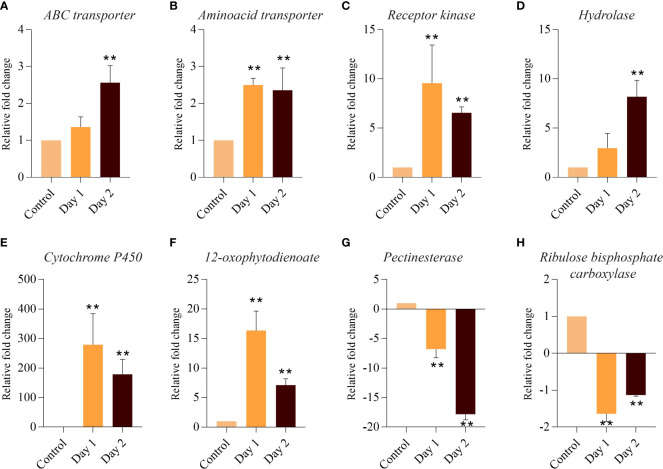
Quantitative PCR-based validation of DEGs associated with coleoptile senescence in rice. Relative expression of genes belonging to functional categories such as transporters: *ABC transporter* **(A)***, amino acid transporter* **(B)**; signaling: *Receptor kinase* **(C)**; hormone signaling and transduction: *Hydrolase* **(D)**, *cytochrome P450* **(E)** and *12-oxophytodienoate* **(F)**; cell wall: *Pectinesterase* **(G)**; and photosynthesis: *Ribulose bisphosphate carboxylase* **(H)** was determined in control and at day 1 and day 2 of senescing coleoptiles. For normalization *eEF-1α* was used as an internal control. Three biological replicates and two technical replicates were included in the study. Asterisks indicate a significant difference between the control and the senescence sample (Student’s *t*-test, **P < 0.01).

RNA-seq derived expression data was reconfirmed with the help of qRT-PCR by examining the expression of 8 DEGs. Relative expression of genes belonging to functional categories such as transporters (*amino acid, ABC transporter*), signalling (*receptor kinase*), hormone signalling and transduction (*hydrolase, cytochrome P450* and *12-oxophytodienoate*), cell wall (*pectinesterase*), and photosynthesis (*ribulose bisphosphate carboxylase*) was determined in control and at day 1 and day 2 of senescing coleoptiles. Both transporters and hydrolase genes showed consistent upregulation in day 1 and day 2 of senescence (**Figures 5A, B, D**). Expression of receptor kinase, cytochrome P450 and 12-oxophytodienoate increased massively on day 1, followed by a slight decline (although upregulated) on day 2 (**Figures 5C, E, F**). Both pectinesterase and ribulose bisphosphate carboxylase showed significant downregulation during progression of senescence. However, pectinesterase exhibited a more severe decline in expression levels on day 2 as compared to ribulose bisphosphate carboxylase (**Figures 5G, H**). The expression patterns of the genes by qPCR analysis were consistent with that of the RNA-sequencing results.

### Identification, digital expression profiling and validation of conserved and novel miRNAs during coleoptile senescence

**Figure 6:**
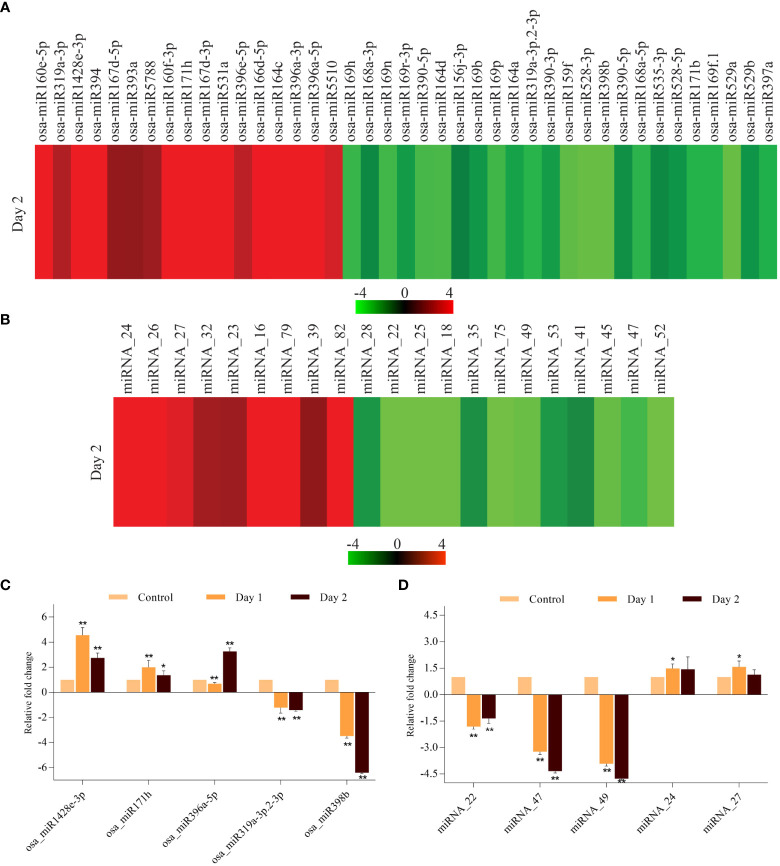
Expression profiling of miRNAs that are differentially expressed during coleoptile senescence in rice. Heat map of differentially expressed miRNAs belonging to ‘conserved miRNAs’ **(A)** and ‘novel miRNAs’ **(B)** categories during coleoptile senescence in rice. Quantitative PCR-based validation of few conserved **(C)** and novel **(D)** miRNAs. For normalization, 5S ribosomal RNA was used as internal control. Three biological replicates and two technical replicates were included in the study. Asterisks indicate significant differences between the control and the senescence sample (Student’s *t*-test, *P < 0.05, **P < 0.01).

Small RNAs are known regulators of gene expression in diverse biological processes in plants such as growth, development and response to biotic and abiotic stresses (Li et al., 2017). The role of miRNAs in regulating induced and natural senescence in leaves has been demonstrated by several studies (Huo et al., 2015; Kim et al., 2018), however, no miRNAs have been identified which have a potential role in controlling the progression of senescence in coleoptile, which is the first organ to senesce in cereals. Therefore, small RNA libraries of control and senescing coleoptiles were sequenced and the data so obtained was analyzed. Scatter plots for comparing small RNA expression pattern between the two biological replicates of control and day 2 samples showed good correlation (**Supplementary Figures 1C, D**). Our analysis identified 66 known miRNAs belonging to thirty-one miRNA families and 41 true novel miRNAs (with star sequences) in the small RNA dataset (**Supplementary Table 6**). For finding the miRNAs that could potentially regulate coleoptile senescence in rice, digital expression levels were analyzed and the miRNAs exhibiting ±2-fold change (log2 scale; average of two biological replicates) in senescing coleoptiles were considered as ‘differentially regulated’. Of the 41 conserved miRNAs, 17 showed upregulation while 24 exhibited downregulation in senescing coleoptiles (**Figure 6A**; **Supplementary Table 7**). The major upregulated miRNA families were miR396 (3 members), miR167 and miR160 (2 members each). Some of the conserved miRNAs; osa-miR160e-5p, osa-miR160f-3p, osa-miR164c, osa-miR167d-3p, osa-miR171h, osa-miR1428e-3p, osa-miR394, osa-miR396a-3p, osa-miR396a-5p, and osa-miR531a were highly upregulated (>4-fold) during coleoptile senescence (**Figure 6A**; **Supplementary Table 7**). The predominantly downregulated miRNA families were miR169 (6 members) and miR390 (3 members). Expression of nine miRNAs (osa-miR159f, osa-miR164d, osa-miR169h, osa-miR169n, osa-miR169p, oss-miR390-5p, osa-miR398b, osa-miR528-3p, and osa-miR529a) declined considerably (more than 3-fold) during coleoptile senescence (**Figure 6A**; **Supplementary Table 7**).

Similar to the conserved miRNAs, novel miRNAs were also regulated during coleoptile senescence. A total of 21 novel miRNAs were differentially expressed, of which 9 miRNAs were upregulated and 12 miRNAs exhibited downregulation (**Figure 6B**; **Supplementary Table 7**). Among the upregulated novel miRNAs, miRNA_16, miRNA_24, miRNA_26, miRNA_79, and miRNA_82 showed more than 4-fold upregulation. Further, greater than 3-fold downregulation was observed in the expression levels of miRNA_18, miRNA_22, miRNA_25, miRNA_45, miRNA_47, miRNA_49, miRNA_52 and miRNA_75 (**Figure 6B**; **Supplementary Table 7**). Expression of 5 conserved and 5 novel miRNAs was validated by poly(T) adaptor RT-PCR method. It was observed that expression of osa_miR1428e-3p and osa_miR171h elevated on day 1 and although their levels declined slightly on day 2, they remained upregulated (**Figure 6C**). On the other hand, levels of osa_miR396c-5p were lower on day 1, but increased rapidly on day 2 of senescence (**Figure 6C**). Among the downregulated conserved miRNAs, osa_miR398b exhibited significantly higher decline than osa_miR319a-3p.2-3p during senescence (**Figure 6C**). The expression of novel miRNAs, miRNA_24 and miRNA_27 increased moderately, while miRNA_47 and miRNA_49 downregulated significantly during coleoptile senescence (**Figure 6D**). Notably, the expression pattern of conserved and novel miRNAs as observed by qRT-PCR was similar with their digital expression profiles.

### Comparison of coleoptile and rice flag leaf senescence miRNAs

To gain insights into the conservation or differences in the miRNA-dependent regulation of senescence in coleoptile and flag leaf, expression of 66 conserved miRNAs was compared with the 38 miRNAs previously identified by our group in flag leaf senescence (FLS) (Sasi et al., 2019). The analysis revealed that 17 conserved miRNAs were detected in both CS and FLS (**Supplementary Table 8**). While 3 conserved miRNAs (osa-miR164a, osa-miR171b, osa-miR535-3p) showed similar expression patterns, 6 miRNAs (osa-miR1428e-3p, osa-miR160e-5p, osa-miR168a-5p, osa-miR171h, osa-miR1432-5p, and osa-miR1861e) displayed an inverse expression pattern in CS and FLS datasets. The remaining 8 miRNAs did not display any remarkable comparative pattern during the two senescence programs. We further compared the expression trends of the 41 true novel miRNAs in CS with 203 true novel miRNAs in FLS and found that 3 miRNAs [(miRNA_2, 24 and 30 which were named as N_osa_619, N_osa_595 and N_osa_176, respectively, in Sasi et al. (2019)] were present in both the datasets. Out of the 3 commonly present novel miRNAs, miR_30 was slightly downregulated in both CS and FLS, while miR_23 was slightly downregulated in FLS, but was upregulated in CS. miR_2 exhibited no significant change in expression level in FLS, however, it was downregulated in CS. The comparative study led to the identification of few miRNAs which probably comprise the core regulatory pathway of senescence programs in rice. It would be worthwhile to determine the expression of their putative targets in the senescence datasets to identify the functionally conserved miRNA-mRNA modules regulating senescence in different tissues of rice.

### Computational prediction of targets of miRNAs, their GO analysis and expression profiling-based validation of selected targets

To elucidate the biological function of miRNAs and other small RNAs it is pertinent to identify the genes targeted by them. The information generated is utilized to unravel the complex regulatory network of miRNA-target interaction and their possible role(s) in regulating different biological processes. We predicted 301 targets for 62 miRNAs (41 conserved and 21 true novel) that were differentially expressed during coleoptile senescence in our dataset (**Supplementary Table 9**). Targets could not be predicted for four conserved (osa-miR169b, osa-miR390-3p, osa-miR394 and osa-miR5788) and one novel (miRNA_47) miRNAs. Except osa-miR1428e-3p, osa-miR169f.1, osa-miR169h, osa-miR319a-3p.2-3p, osa-miR396a-3p, osa-miR5510 and miRNA_23 which were predicted to hit only a single target gene, all other miRNAs were predicted to target multiple genes. The maximum number of targets were 44 for osa-miR531a, followed by 14 for osa-miR156j-3p. Gene ontology clustering analysis of the predicted targets revealed that the maximum number of targets were associated with molecular function (207) followed by biological process (145) (**Supplementary Table 9**). The top sub-categories in each gene function cluster belonged to ATP-binding, transferase activity, membrane proteins, protein phosphorylation, oxidation-reduction process and regulation of transcription. All these observations indicate that these miRNAs might regulate diverse processes in coleoptile senescence program.

**Figure 7:**
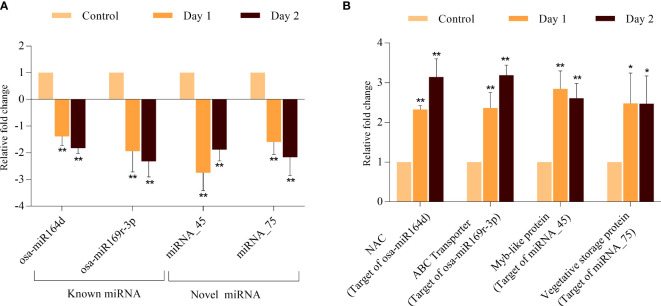
Validation of expression profile of miRNAs and their predicted target genes during coleoptile senescence in rice. Expression analysis of selected miRNAs **(A)** and one of their predicted targets genes **(B)** using qPCR. For normalization of real-time PCR results, *eEF-1α* was used as internal control. Three biological replicates and two technical replicates were included in the study. Asterisks indicate significant differences between the control and the senescence sample (Student’s *t*-test, *P < 0.05, **P < 0.01).

To validate the predicted regulatory relationship between the senescence-associated miRNAs and their target, we performed qRT-PCR of osa-miR164d, osa-miR169r-3p, miRNA_45 and miRNA_75 and their corresponding putative target genes. The expression patterns of all miRNAs and their targets exhibited consistency with the digital expression data obtained in RNA-seq and small RNA-seq experiments (**Figure 7**). osa-miR164d was predicted to target OsNAC2 or OsORE1 (encoded by LOC_Os04g38720) which was significantly upregulated during progression of coleoptile senescence. Similarly, osa-miR169r-3p exhibited downregulation and its target ABC transporter (encoded by LOC_Os11g07600) accumulated to high levels. One of the targets of novel miRNA_45 was MYB TF encoded by LOC_Os05g07010 whose expression levels were upregulated. The expression profiling analyses of miRNA-mRNA interactions indicated the high confidence level of the prediction of the targets and the requirement of testing more such pairs to gain an insight into the regulation of coleoptile senescence in rice.

### Regulatory network of miRNAs and their target genes

**Figure 8:**
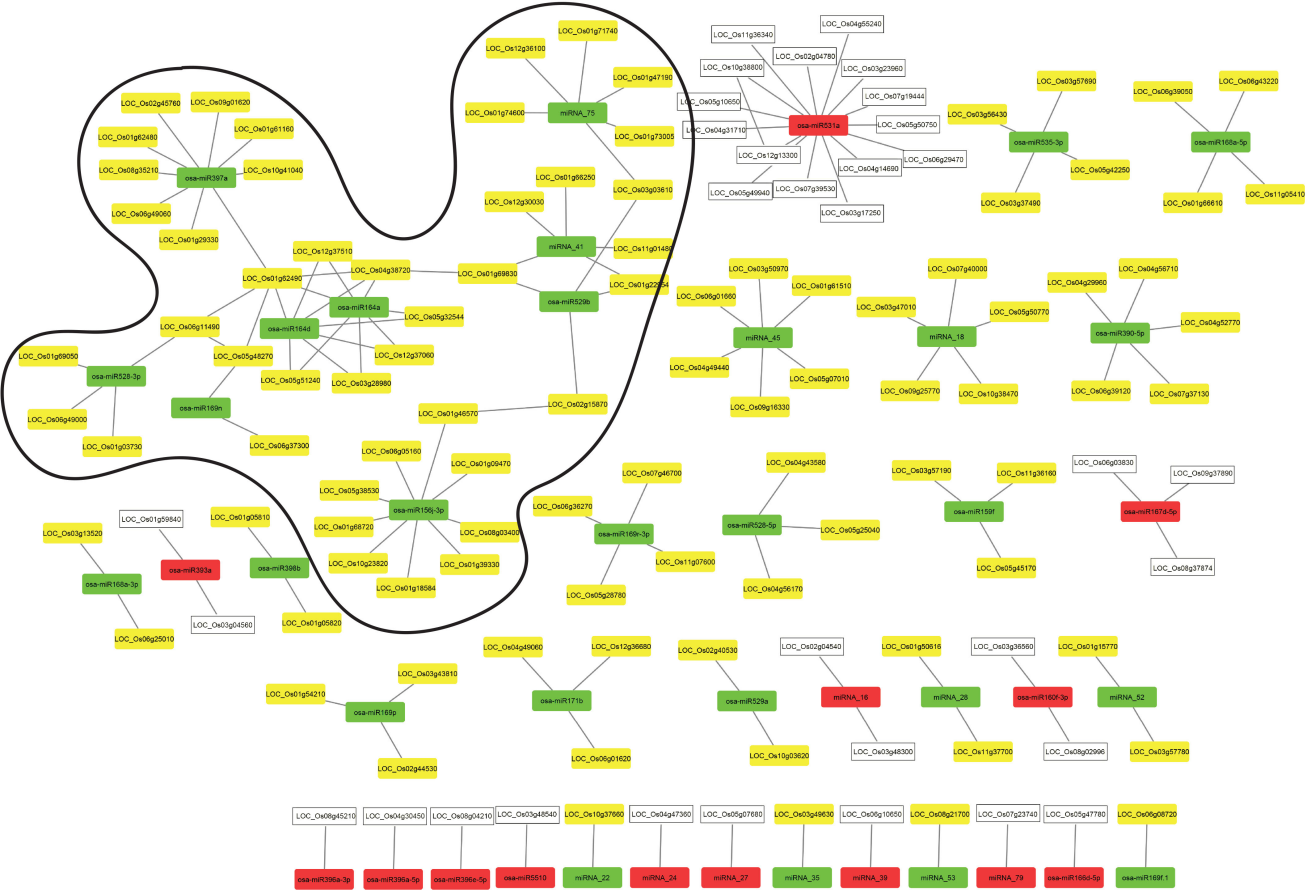
Integration of regulatory miRNA-mRNA network with PPI network during coleoptile senescence in rice. Protein-protein interaction network for the predicted target genes of conserved and novel miRNAs was generated using Cytoscape visualization software. The expression of miRNAs and target genes is presented based on the RNA-seq and small RNAome data obtained for coleoptile senescence in rice, respectively. Upregulated and downregulated miRNAs are represented by red and green boxes, respectively. Upregulated and downregulated predicted target genes are represented by yellow and white boxes, respectively. The predominant hub identified in the present study is highlighted.

To identify the key regulatory components and their interactors in coleoptile senescence, a combinatorial approach was employed to integrate the regulatory dataset of RNA-seq and small RNA-seq with publicly available protein-protein interaction (PPI) dataset (Szklarczyk et al., 2021). Expression of putative miRNA-targets was searched in the transcriptome data and the target genes whose expression correlated inversely with that of corresponding miRNAs were interrogated for their protein interactions (**Supplementary Tables 9**, **10**). The PPI analysis output was used to generate miRNA-target regulatory networks using the Cytoscape software (**Figure 8**). It was noted that 105 known miRNA-target pairs and 35 novel miRNA-target pairs showed an inverse correlation in the expression profile (**Figure 8**). The biological regulatory network model consisted of 14 and 28 modules composed of upregulated and downregulated miRNAs targeting the genes that exhibited significant decline and elevation in expression level during coleoptile senescence, respectively (**Figure 8**). An important module was controlled by an upregulated miR531a whose all the 15 target genes exhibited decline in their expression levels during senescence. Among these 15 targets, two genes encoded for LRR transmembrane protein kinase and an expressed protein and they are known to interact which has been validated experimentally (Szklarczyk et al., 2021). It would be worthwhile to validate the targets and identify which of these contribute to coleoptile senescence. Similarly, other downregulated conserved miRNAs such as miR397a and miR156j could target 9 genes each, while miR164a was found to target 7 genes, expression of all of which was suppressed during senescence. Two novel miRNAs, miRNA_45 and miRNA_75 were predicted to target 6 genes each and these genes also exhibited an inverse correlation in expression. Majority of the modules in the regulatory network consisted of senescence-repressed miRNAs and their corresponding senescence-upregulated targets and therefore protein-protein interactions were largely predicted within these modules.

At the center of the predominant hub were two members of the MIR164 family, miR164a and miR164d. The sequences of miR164a and miR164d are similar except that miR164a had an extra nucleotide at its 3’ end. However, this did not affect the ability of these miRNAs to target the same genes (**Supplementary Tables 9**, **10**). Interestingly, proteins coded by two of their targets i.e. LOC_Os01g62490 (coding for a laccase precursor) and LOC_Os04g38720 (a NAC TF), appeared to be interacting with each other. These proteins further served as connectors with four additional modules; a) NAC protein interacted with OsSPL2 protein encoded by LOC_01g69830 (a target of miRNA_41 and osa-miR529b; b) laccase precursor interacted with a plastocyanin-domain containing protein encoded by LOC_06g11490 (a target of osa-miR528-3p); c) laccase precursor is also a target of osa-miR397a, thereby connecting the osa-miR164 and osa-miR397a nodes; d) laccase precursor interacted with an auxin-responsive protein encoded by LOC_Os05g48270 (a target of osa-miR169n). Additional connections between different modules were seen such as the Molybdenum cofactor biosynthesis protein 1 encoded by LOC_Os02g15870 (target of osa-miR529b) interacted with CTP synthase protein encoded by LOC_Os01g46570 (target of osa-miR156j-3p). Intermodular connection was also observed between the module regulated by miRNA_75 and module regulated by miRNA_41 and osa-miR529b, wherein 1,3-beta-glucan synthase component domain containing protein (encoded by LOC_Os03g03610) was a target of both osa-miR529b and miRNA_75. Intramodular connections were seen in the module regulated by miRNA_41 and osa-miR529, both of which shared two targets i.e., LOC_Os01g69830 and LOC_01g22954. Only few interactions among different proteins encoded by miRNA-targeted genes in the dataset were observed. However, most of the PPIs identified in the network are predicted on the basis of their co-expression or database or text mining. It is, therefore, necessary to validate these interactions experimentally and then characterize their role in coleoptile senescence in rice. It is expected that with the enrichment of PPI data in the public domain, more connectivity between the modules (both inter-modular and intra-modular) will be observed which would highlight the functional and dynamic complexity of proteins and miRNA regulation of their target genes.

## Discussion

Senescence is a precise and highly orchestrated degradation process that is essential for reallocation of resources to developing organs and overall fitness of the plants. The genetic complexity and molecular mechanisms underlying natural leaf senescence have been well-studied, however, owing to slow progression of flag leaf senescence and long experimental duration with flag leaf being the last leaf to emerge, studying flag leaf senescence is challenging. On the other hand, coleoptile the first organ to senesce after seed germination in monocotyledonous plants undergoes a rapid transition from growth to death phase and due to which there is more uniformity in the coleoptile senescence as compared to that of leaves (Kawai and Uchimiya, 2000). Nevertheless, the key changes at tissue and cellular levels associated with senescence in coleoptiles and leaves are conserved (Inada et al., 2002). The unique features of coleoptile senescence thus advocates the possibility of its utilization as a convenient and rapid system for studying the cellular and molecular mechanisms of senescence in cereals. With an aim to unravel the functional biological networks of coleoptile senescence in rice, an integrative multi-omics approach was designed which combined high-throughput RNA-seq and small RNA-seq data of the senescing coleoptile generated in the present study with publicly available protein-protein interaction (PPI) datasets.

Our study reports identification of 3166 differentially expressed and 273 senescence-specific genes (jointly referred to as DEGs) in rice coleoptile senescence. Several of these DEGs encodes for transporter proteins, more interestingly, proportion of upregulated transporters (112) was nearly 5 times higher than those exhibiting downregulation (22). Transport of ions and other macromolecules across cellular boundaries is a key event that ensures remobilization of nutrients from source to sink (Guo et al., 2021). Previous studies on elucidating the molecular changes occurring during leaf senescence reported an increased expression of genes encoding for several transporters, especially ABC and MATE type, amino acid and ion transporters (Buchanan-Wollaston et al., 2005; Graaff et al., 2006; Lee et al., 2017). ABC, peptides, amino acids, metabolites, sugar, and metal transporters were abundantly expressed during CS, which is in agreement with earlier reports (Guo et al., 2004; Zhang et al., 2014). Among the upregulated transporters, 11 genes each for MATE (Multidrug And Toxic compound Extrusion) and aquaporins (AQPs) were identified. MATE protein family in rice has 46 members and are involved in transport of secondary metabolites, ions and phytohormones and are therefore associated with regulation of several agronomic traits by mediating growth, development and response to environmental stimuli (Upadhyay et al., 2019; Du et al., 2021). Overexpression of *ELS1* (a MATE transporter) in Arabidopsis accelerated the senescence (Wang et al., 2016). Further, ABS3 (abnormal shoot 3), a MATE transporter protein, interacts with ATG8 (autophagy-related protein 8) and promotes senescence under natural and carbon-deprivation conditions (Jia et al., 2019). Functional characterization of the differentially expressed MATE transporters will help in identifying the transported molecules during CS. Another transporter family which attracted our attention was aquaporins (AQPs), which are expressed under a myriad of physiological conditions and regulate plant development and stress in plants (Maurel et al., 2008). These channel proteins are actively involved in water transport and trafficking of small solutes such as silicon, urea, reactive oxygen species (ROS) and even gasses such as CO_2_ and ammonia (Maurel et al., 2008). Interestingly, CS involved upregulation and downregulation of 11 and 1 aquaporins, respectively. Functionally, AQPs are involved in plant senescence as the suppression of TIP1;1, a member of tonoplast AQPs, compromised carbohydrate transport, had excessive water loss and accelerated leaf senescence (Ma et al., 2004), while overexpression of the cotton TIP1;1-like protein in Arabidopsis promoted premature bolting and delayed senescence of rosette leaves (Cheng et al., 2022). AQPs promote diffusion of H_2_O_2_ across the membranes during senescence which may act as a signal for triggering senescence in the adjoining cells (Zentgraf et al., 2022). During senescence, generation of ROS, especially H_2_O_2_ is one of the early responses and it acts as a potent signaling reactive oxygen species. H_2_O_2_ further reprograms expression of AQPs and other SAGs. In addition to regulating the expression, H_2_O_2_ is also believed to modulate the AQPs transportation efficiency, structure and localization (Luu and Maurel, 2005). Owing to the multitasking roles of different transporters in plants, it would be worth exploring the function of these transporters in plant senescence.

Members of TF families such as NAC, MYB, WRKY, TCP, AP2/EREBP, bZIP and bHLH are associated with senescence (Guo et al., 2004; Gregersen and Holm, 2007; Breeze et al., 2011; Zhang et al., 2014; Lin et al., 2015; Moschen et al., 2016; Hinckley and Brusslan, 2020; Dong et al., 2021). The number of upregulated members of TF families AP2/EREBP, Zinc finger, homeobox, MYB, bHLH and WRKY TFs was greater than the down regulated members during CS, thus conforming to the previous observations (Zhang et al., 2014; Lin et al., 2015). Heat shock transcription factors (HSFs) have been indirectly associated with senescence and an early senescence phenotype was reported in the knock-out mutant of *AtHSFB1* in Arabidopsis (Breeze et al., 2008). Wu et al. (2012) demonstrated that JUNGBRUNNEN1 (JUB1), a NAC TF, functions as a negative regulator of senescence through its action on HSFA3 which elevates accumulation of HSPs and lowers H_2_O_2_ levels (Wu et al., 2012). Our results indicate that HSF family members play a more crucial role in regulating senescence as 5 members of HSF family (*OsHSFA2b, OsHSFA3a, OsHSFA7a, OsHSFB2b* and *OsHSFC1b*) were significantly upregulated during CS. Comprehensive expression profiling of HSFs has also revealed differential regulation of several HSFs in natural and induced flag leaf senescence in rice (Sasi et al., unpublished). Further, we found that the putative promoter regions of approximately 5% of SAGs in rice possess canonical and non-canonical heat shock elements (HSEs), thus indicating that HSFs might regulate the expression of SAGs by binding to their promoters (Sasi et al., unpublished).

Phytohormone homeostasis genes, together with an active involvement of regulatory factors such as TFs and small RNAs, fine tune senescence program in plants by integrating the developmental and environmental signals (Staden, 1995; Schippers et al., 2007; Khan et al., 2014). We observed exclusive upregulation for several SA and JA pathway genes. Additionally, the proportion of upregulated genes associated with cytokinin metabolism were largely downregulated, but those related with the auxin metabolism were upregulated. The levels of cytokinins decline during leaf senescence and exogenous application of cytokinins delay senescence (Guo et al., 2021). Although, upregulation of a few IAA (indole acetic acid) biosynthesis genes during leaf senescence has also been previously observed (Quirino et al., 1999), auxin also appears to play a negative role by suppressing expression of WRK57 and its downstream target SAG12, thereby delaying senescence in Arabidopsis (Noh and Amasino, 1999). A comparative transcriptome study revealed that SA, JA and ethylene are predominantly associated with developmental and dark-induced leaf and cell-suspension senescence, respectively (Buchanan-Wollaston et al., 2005). Transcript profiling of hormone pathway genes in senescing leaves in Arabidopsis and cotton demonstrated the involvement of several hormones such as salicylic acid (SA), ethylene, auxin, jasmonic acid and cytokinin, ABA and BR (Graaff et al., 2006; Lin et al., 2015). BRs accelerate senescence in detached cotyledons and leaves in wheat and pea (Sağlam-Çağ, 2007; Fedina et al., 2016) and acts synergistically with auxin to induce senescence in soybean cotyledons (Baris and Saglam-Cag, 2016). ABA enhances plant senescence by positively regulating the expression of various chlorophyll degradation genes (*NON-YELLOW COLORING1, STAY-GREEN, PHEOPHYTINASE* and *PHEIDE A OXYGENASE*) and NAC TF family members (*VND-INTERACTING2 (VNI2), A SUBFAMILY OF STRESS-RESPONSIVE NAC (SNAC-A), ORESARA1 (ORE1), NAC-LIKE ACTIVATED BY APETALA3/PISTILLATA (OsNAP)* and *OsNAC2*) (Pruzinská et al., 2005; Kusaba et al., 2007; Park et al., 2007; Schelbert et al., 2009; Kim et al., 2011; Yang et al., 2011; Liang et al., 2014; Takasaki et al., 2015; Mao et al., 2017).

Unfavorable growth conditions such as salinity, extreme temperatures, dehydration, oxidative and nutrient deprivation induce premature senescence in plants. In fact, gene expression analyses have shown that several stress-responsive genes, including regulatory genes, are induced during progression of senescence (Breeze et al., 2011; Dong et al., 2021; Li et al., 2021). It is, therefore, believed that the molecular pathways of leaf senescence cross-talk with stress responsive pathways in plants (Guo and Gan, 2012). Our study revealed that several PR genes, conventionally associated with biotic stress, were differentially expressed in senescing coleoptiles, which is consistent with the previous studies where PR protein encoding genes were categorized as SAGs (Barth et al., 2004; Sillanpää et al., 2005). Accumulation of PR proteins was also observed in the intercellular spaces of barley leaves during natural senescence (Tamás et al., 1998). Protein profiling of apoplastic proteins of naturally, but not dark-induced, senescing leaves of Arabidopsis revealed that the most abundant SAG proteins were PR2 and PR5 (Borniego et al., 2020). Pathogens interfere with the host development by modifying the signalling pathways and regulating progression of plant senescence to meet their nutritional demands, whereas plants counteract by inducing a hypersensitive response to kill its own cells. When plants are challenged by a pathogen, induction of PR proteins along with several TFs is necessary for triggering the defense-related responses (Häffner et al., 2015). It would be worth comparing the induction patterns of PR genes in genotypes with contrasting senescence timing as well as correlating the progression of senescence with steady state levels of PR genes in pathogen-resistant versus susceptible genotypes. It is, therefore, pertinent to understand how the complex interaction of host and pathogen triggers senescence so as to pinpoint the cross-talk, signaling branches and regulatory nodes that play crucial roles in both pathogen response and senescence. Under abiotic stress category, heat stress-related genes such as DnaJ-related heat shock proteins (HSPs), several HSP70s, small HSPs (HSP18.2, HSP17.4, mitochondrial HSP23) exhibited significant upregulation during senescence. Although the potency of heat shock response (HSR) declines at the terminal stages of development, several components of HSR accumulate specifically during aging (Taylor and Dillin, 2011). One class of such proteins is molecular chaperones, which prevent protein aggregation observed during senescence and stress (Calderwood et al., 2009; Trivedi and Jurivich, 2020). Induction of HSFs and HSPs during stress and senescence thus indicate the existence of cross-talk between the senescence and abiotic stress pathways.

Although senescence of coleoptile and flag leaf supplies nutrition to growing seedlings and developing seeds respectively, the overall physiology of senescence in these tissues is vastly different. Whereas coleoptile emerges from the seed and exhibits rapid senescence, second leaf and flag leaf emerge from photosynthesizing plants and have a slow progression of senescence. To explore the extent of similarity and highlight the core components involved in senescence of two spatially and temporally different organs in rice (coleoptile and leaves) DEGs of CS and LS were compared. We observed that approximately 92% of the DEGs had different expression profiles in CS and LS, which is a reflection of their divergent physiologies at the level of gene expression. Among the 8% genes that were similarly regulated, 3 AQP-coding genes were commonly induced during both leaf and coleoptile senescence, however, overrepresentation of upregulated AQPs in CS (11) as compared to LS (4) also highlight specific roles of the aquaporins during coleoptile senescence. It would be worth performing additional functional studies to understand the convergent and divergent roles that the common and specific AQPs play during senescence in rice. Among the TFs, few members such as *WRKY*s, *MYB*s, *NAC*s and *Zinc finger proteins* along with 3 HSFs (*OsHSFA3a*, *OsHSFA7a* and *OsHSFC1b*) were commonly upregulated in CS and LS. The roles of transporters and TFs during plant senescence have been discussed previously in this article. Since AQPs, HSFs and other TFs are also involved in stress-responsive pathways, stress and aging pathways appear to be closely related.

Several genes encoding ELIPs (early light-induced proteins) were also upregulated in both CS and LS. ELIPs bind to free chlorophyll released from pigment-protein complexes and provide protection against oxidative stress during senescence (Binyamin et al., 2001; Hutin et al., 2003). Similarly RCCR (red chlorophyll catabolite reductase) induced in both types of senescence is involved in chlorophyll degradation, an integral component of plant senescence (Pruzinská et al., 2005). Another important gene associated with the initial step of the chlorophyll degradation pathway, *OsSGR* (stay-green), was also expressed at high levels during senescence in coleoptiles and flag leaves in rice. OsSGR encodes a vital enzyme, magnesium (Mg) dechelatase that removes Mg from chlorophyll-a resulting in its conversion to pheophytin A (Phein A), an intermediate in the universal chlorophyll breakdown pathway (Shimoda et al., 2016). Furthermore, SGR binds to LHCII and accelerates the breakdown of chlorophyll-protein complexes (Sakuraba et al., 2012). Several CYPs (cytochrome P450 monooxygenases) exhibited upregulation in both CS and LS. CYPs are known as ‘versatile biocatalysts’ because of their involvement with biosynthesis of antioxidants, secondary metabolites and phytohormones (Pandian et al., 2020), whose levels change with progression of senescence. The role of cytochrome P450 members in brassinosteroid signaling is well-documented in the studies on rice dwarf mutant, *dwarf11* (Tanabe, 2005). Cell wall breakdown and a decline in photosynthesis are often associated with the senescence process (Mohapatra et al., 2010) and we also observed that several genes belonging to the cell wall such as pectinesterase and photosynthesis-related categories such as *Ribulose bisphosphate carboxylase small chain precursor*, displayed significant decline in expression levels. Pectinesterase is involved in cellular adhesion and stem elongation (Li et al., 2017) and its level decreases in senescing strawberries (Castillejo et al., 2004). Ribulose bisphosphate carboxylase small chain precursor is the core component of photosynthesis (Bannenberg et al., 2009) and systematic degradation of chlorophyll during senescence could possibly be related to the decreased expression of Ribulose bisphosphate carboxylase small chain precursor. One and two genes encoding for 1-aminocyclopropane-1-carboxylate oxidase (ACO) family were upregulated and downregulated, respectively, during both CS and LS. ACO members are differentially expressed developmentally and in tissue-specific manner (Houben and Van de Poel, 2019) and regulate various developmental processes and stress responses in plants (Houben and Van de Poel, 2019). Since ACOs exert a precise control on spatial and temporal levels of ethylene it will be interesting to correlate change in expression of individual ACO members with ethylene levels and senescence programs.

In addition to the genes that were co-expressed, multiple genes showed an inverse expression profile during the two senescence programs. GO enrichment revealed that the top category of genes belonged to the ‘integral component of membrane’ followed by DNA binding/transcription. Additionally, several TFs belonging to the MYB family, CYPs, GSTs (glutathione-S-transferase), LTPLs (lipid transfer proteins), glycosyl hydrolases, transporters and stress-responsive proteins exhibited differential expression patterns during progression of senescence in two tissues. We believe that finding the downstream constituents of the inversely correlated genes will help in finding the basis of the different physiologies seen in these senescence programs. An important gene involved in ABA synthesis, i.e., *NCED1* encoding for 9-cis-epoxycarotenoid dioxygenase accumulated in CS, while its levels declined in LS. Modulating expression of NCED genes can affect the timing of senescence e.g., overexpression of *OsNCED3*, and *OsNCED5* genes that are strongly induced by dark, accelerate leaf senescence in rice (Huang et al., 2018; Huang et al., 2019). NCED1 is a rate-limiting enzyme in the ABA biosynthesis pathway and since ABA is a positive regulator of senescence in plants, upregulation of NCED1 explains the positive correlation between the NCED1 expression and CS. However, the reason for its downregulation in LS is not known. The downregulation of NCED1 in LS was observed by comparing the expression levels at 36 days post heading (DPH) with 4 DPH stage and it is possible that NCED1 levels elevate at a later stage of senescence in flag leaves. The effect of modulating the expression levels of other members of NCED gene family on natural and dark-induced senescence in rice is not known and worth exploring. Since ABA is synthesized from β-carotene, we analyzed the expression pattern of carotenoid biosynthesis genes in CS as well. At least 16 genes involved in carotenoid production have been identified in rice (Chaudhary et al., 2010), of which two genes, *Phytoene synthase* (LOC Os09g38320) and *Lycopene є-cyclase* (LOC Os01g39960), were found to be up-regulated in our dataset. During α-carotene biosynthesis, lycopene є-cyclase catalyzes the cyclization of linear lycopene to produce cyclic lycopene (Bai et al., 2009). Phytoene synthase is the major rate-limiting enzyme in the carotenoid biosynthesis pathway (Simpson et al., 2018). It is well documented that ABA can induce Phytoene synthase to activate the production of its precursors (Simpson et al., 2018). This suggests that phytoene synthase and carotenes may participate in an auto-activating ABA biosynthesis pathway during CS in rice.

Small RNAs play an important role in regulating gene expression during growth and development and response to different stresses in plants (Li et al., 2017). A number of miRNAs associated with regulation of leaf senescence have been identified in multiple plant species (Xu et al., 2014; Huo et al., 2015; Thatcher et al., 2015; Qin et al., 2016; Wu et al., 2016; Swida-Barteczka and Szweykowska-Kulinska, 2019; Sasi et al., 2019). However, the contribution of miRNAs in controlling coleoptile senescence has not been elucidated so far. Therefore, in the present study miRNAs that are differentially expressed during progression of senescence in coleoptiles and their target genes were predicted. Further miRNAs and their corresponding target genes which exhibited an inverse expression pattern during coleoptile senescence were identified. Overall, 41 conserved and 21 novel miRNAs exhibiting differential expression patterns in senescing coleoptiles were identified. To find out the commonality in the senescence programs of different tissues of rice, we compared the miRNAs identified in the CS and flag leaf senescence (Sasi et al., 2019) and found that 3 miRNAs were commonly downregulated in both datasets. Interestingly, when the expression pattern of the predicted target genes of these miRNAs was determined in CS (present study) and FLS (Lee et al., 2017) RNA-seq datasets, we found that the targets of 2 commonly downregulated miRNAs (osa-miR164a: LOC_Os04g38720 which encodes for NAC2 and osa-miR535-3p: LOC_Os05g42250 which encodes for cyclic nucleotide-gated ion channel 2 or OsCNGC16) exhibited remarkable upregulation which indicates that these miRNA-mRNA pairs could play an important role in plant senescence. OsCNGC16 is a plasma-membrane localized protein which triggers an increase in calcium levels when plants are exposed to extreme temperatures (heat or chilling) and imparts thermotolerance in rice (Cui et al., 2020). Calcium also plays an important role in triggering senescence in plants as exogenous application of calcium delays senescence in detached leaves (Poovaiah and Leopold, 1973). The importance of calcium in senescence is further evident by the action of calcium-dependent protein kinase 1 (CPK1) in phosphorylating ORE, the master regulator of senescence in Arabidopsis (Durian et al., 2020). It would be interesting to validate these miRNAs and their respective targets in different senescence tissues and perform their functional characterization for possible involvement in regulation of senescence in plants.

Integration of expression datasets of RNA-seq and small RNA-seq of coleoptile senescence unraveled the important miRNA-mRNA modules and provided a thorough understanding of the associated multiple regulatory networks. Only high confidence miRNA-target pairs which exhibited inverse expression patterns in miRNAome and transcriptome datasets generated by us, respectively, were utilized for the construction of regulatory network. Further, publicly available protein-protein interaction (PPI) data was also integrated with the miRNA-mRNA network and visualization of regulatory network unravelled 140 miRNA-mRNA modules, while only 6 protein interactions were identified. Based on the network generated we concluded that:

- A predominant hub consists of 9 upregulated miRNAs, including two members of MIR164 family at the center, targeting 42 genes during coleoptile senescence. Previous studies have reported few of these reliable miRNA-target pairs in rice and other plant species: osa-miR164a/d-NAC2, osa-miR397a-laccase, osa-miR528-plastocyanin, osa-miR529b-SPL2 (Fang et al., 2014; Pan et al., 2017; Tsuzuki et al., 2019; Zhu et al., 2020). Some of these miRNAs such as osa-miR164, osa-miR528 and their targets genes NAC2 and COPPER ION BINDING PROTEIN1 have been implicated in regulation of leaf senescence (Yuan et al., 2015; Mao et al., 2017). SPLs are also associated with regulation of flowering time, phase transition and developmental aging (Jung et al., 2016).
- The coregulatory miRNA-target pairs are connected by the characteristic nature of miRNAs targeting more than one gene or one gene targeted by several miRNAs. For example: While osa-miR164a/d target NAC2 and laccase, laccase is also a target of osa-miR397a. SPL2 and serine carboxypeptidase are targets of both osa-miR529b and miRNA_41. 1,3 beta glucan synthase, being the common target of osa-miR529b and miRNA_75, links the two miRNA modules. The autolysis of cellular components during PCD has been linked to serine carboxypeptidases (Schaller, 2004). However, no reports were found that directly implicate 1,3 beat glucan synthase and laccases in plant senescence.
- Several targets of miRNAs are TFs which regulate diverse biological processes at different stages of plant development and few of these have been implicated in regulation of senescence such as WRKY, NAC, SPL, MYB, TCP, and F-box protein as discussed earlier (Woo et al., 2001; Schommer et al., 2008; Zhang et al., 2011; Jiang et al., 2014; Liang et al., 2014; Jung et al., 2016).
- miR169n is predicted to target two genes, LOC_Os05g48270 (DOMON domain-containing protein-cytochromeb561/ferric reductase transmembrane protein/auxin responsive protein) and LOC_Os06g37300 (cytochrome P450 or CYP). In rice, CYPs belong to a large multigene family, the members of which are associated with metabolism of fatty acids, phenylpropanoids and steroids and more importantly, with hormone homeostasis, thereby regulating plant development and stress responses (Wei and Chen, 2018). A recent report demonstrated that a mutant of *ELL1* which encodes for CYP monooxygenase displayed high accumulation of ROS and cell death in rice (Cui et al., 2021). DOMON gene was identified as a strong candidate in the QTLs for functional stay-green (FSG) trait in rice (Lim and Paek, 2015). However, it is important to investigate the exact function of LOC_Os05g48270 and how it regulates coleoptile senescence in rice.
- Two copper-containing proteins, laccase (multi-copper) and plastocyanin (mono-copper), are part of the hub wherein one member (LOC_Os01g62490) of laccase family is targeted by three miRNAs, osa-miR164a/d and osa-miR397a, while the two other members (LOC_Os01g61160, LOC_Os01g62480) are targeted by osa-miR397a only. The gene (LOC_Os06g11490) encoding endomembrane plastocyanin is targeted by osa-miR528-3p. In plants Cu-miRNAs such as miR397, miR408, miR528 and miR857 target copper-containing proteins: laccases, plastocyanin, polyphenol oxidase (PPO), ascorbate oxidase (AAO), amino oxidase (AO), Cu/Zn superoxide dismutases (CSDs; Zhu et al., 2020). These Cu-containing proteins are actively involved in diverse processes such as ROS metabolism, Cu homeostasis and stress tolerance (Zhu et al., 2020). In Arabidopsis, a pair of interacting proteins belonging to blue copper protein family: plantacyanin (PCY) and SAG14 are induced during dark-induced senescence which is crucial for chloroplast copper efflux and this module is regulated by miR408 and PIF3/4/5 (Hao et al., 2022). Plant laccases are Cu-oxidases which catalyze lignin and anthocyanin biosynthesis and are involved in diverse plant processes: growth and development, defense against biotic and abiotic stresses (Ranocha et al., 2002; Wan et al., 2022). However, as per our understanding no studies have directly linked laccase with regulation of senescence in plants. In the present study we show that three laccase-encoding genes are upregulated and 3 miRNAs that are possibly regulating their expression are clearly downregulated. Our results highlight the possible involvement of laccases in regulating coleoptile senescence in rice, however, the underlying mechanism needs to be explored. It is hypothesized that the copper-proteins might contribute to regulation of senescence by modulating Cu and redox homeostasis in plants.

## Conclusions

We employed an integrated omics approach by combining transcriptome and small RNAome sequencing of coleoptile senescence in rice. Analysis of the RNA-seq dataset identified 3439 DEGs many of which were TFs, transporters, and involved in hormone metabolism, redox and stress responses. Comparison of CS dataset with publicly available leaf senescence (LS) dataset discovered a set of core senescence associated genes. Small RNA sequencing identified many differentially expressed miRNAs, targets for some of which displayed an inverse expression profile in transcriptome dataset. The regulatory network generated by integrating the high confidence miRNA-RNA target pairs with PPI dataset was comprised of 42 modules. A distinguished hub of miR164a/d targeting copper-containing proteins, laccases and plastocyanin, indicated that copper and ROS homeostasis regulate coleoptile senescence in rice. Overall, our study reports a comprehensive atlas on the expression dynamics of genes, miRNA regulators and their interactions and provides insights into the fundamental pathways regulating coleoptile senescence in rice.

## Data availability statement

The datasets presented in this study can be found in online repositories. The names of the repository/repositories and accession number(s) can be found in the article/**Supplementary Material**.

## Author contributions

SK: Conceptualization, Supervision, Investigation, Formal analysis, Funding acquisition, Methodology, Resources, Validation, Writing-original draft, review and editing; MA: Investigation, Methodology, Resources, Writing-critical review and editing; JMS: Investigation, Methodology, Validation, Data curation, Formal Analysis, Writing-original draft, review and editing; CV: Data analysis, curation and Writing-review and editing; BK: Data analysis and curation; RB: Data curation, formal analysis, software; RS: Data curation, formal analysis, software. All authors contributed to the article and approved the submitted version.

## Funding

The financial support from University Grants Commission (sanction order # 41-512/2012), Government of India; Department of Biotechnology, Government of India and Faculty Research Programme (Institute of Eminence), University of Delhi (sanction orders # IoE/FRP/2020/27 and IoE/2021/12/FRP) are acknowledged. JMS and CV are thankful to UGC for Basic Science Research (BSR) fellowships.

## Conflict of interest

Authors RB and RS are employed by the company Bionivid Technology Pvt. Limited, Bengaluru, Karnataka, India.

The remaining authors declare that the research was conducted in the absence of any commercial or financial relationships that could be construed as a potential conflict of interest.

## Publisher’s note

All claims expressed in this article are solely those of the authors and do not necessarily represent those of their affiliated organizations, or those of the publisher, the editors and the reviewers. Any product that may be evaluated in this article, or claim that may be made by its manufacturer, is not guaranteed or endorsed by the publisher.

## References

[B1] Ay N. Janack B. Humbeck K. (2014). Epigenetic control of plant senescence and linked processes. J. Exp. Bot. 65 (14), 3875–3887. doi: 10.1093/jxb/eru132 24683182

[B2] Bai L. Kim E. H. Dellapenna D. Brutnell T. P. (2009). Novel lycopene epsilon cyclase activities in maize revealed through perturbation of carotenoid biosynthesis. Plant J. 59 (4), 588–599. doi: 10.1111/J.1365-313X.2009.03899.X 19392686

[B3] Bannenberg G. Martínez M. Rodríguez M. J. López M. A. Ponce de León I. Hamberg M.. (2009). Functional analysis of alpha-DOX2, an active alpha-dioxygenase critical for normal development in tomato plants. Plant Physiol. 151 (3), 1421–1432. doi: 10.1104/pp.109.145094 19759339 PMC2773050

[B4] Baris C. Saglam-Cag S. (2016). The effects of brassinosteroids on sequential leaf senescence occurring in glycine max l. Int. J. Bio-Technology Res. (IJBTR) 6 (4), 7–16.

[B5] Barth C. Moeder W. Klessig D. F. Conklin P. L. (2004). The timing of senescence and response to pathogens is altered in the ascorbate-deficient arabidopsis mutant vitamin c-1. Plant Physiol. 134 (4), 1784–1792. doi: 10.1104/pp.103.032185 15064386 PMC419851

[B6] Binyamin L. Falah M. Portnoy V. Soudry E. Gepstein S. (2001). The early light-induced protein is also produced during leaf senescence of nicotiana tabacum. Planta 212 (4), 591–597. doi: 10.1007/S004250000423 11525516

[B7] Borniego M. L. Molina M. C. Guiamét J. J. Martinez D. E. (2020). Physiological and proteomic changes in the apoplast accompany leaf senescence in arabidopsis. Front. Plant Sci. 10. doi: 10.3389/fpls.2019.01635 PMC6960232 31969890

[B8] Breeze E. Harrison E. McHattie S. Hughes L. Hickman R. Hill C.. (2011). High-resolution temporal profiling of transcripts during arabidopsis leaf senescence reveals a distinct chronology of processes and regulation. Plant Cell 23 (3), 873–894. doi: 10.1105/tpc.111.083345 21447789 PMC3082270

[B9] Breeze E. Harrison E. Page T. Warner N. Shen C. Zhang C. (2008). Transcriptional regulation of plant senescence : from functional genomics to systems biology Plant Bio. 10, 99–109. doi: 10.1111/j.1438-8677.2008.00076.x 18721315

[B10] Buchanan-Wollaston V. Page T. Harrison E. Breeze E. Lim P. O. Nam H. G.. (2005). Comparative transcriptome analysis reveals significant differences in gene expression and signalling pathways between developmental and dark / starvation-induced senescence in arabidopsis. Plant J. 42, 567–585. doi: 10.1111/j.1365-313X.2005.02399.x 15860015

[B11] Calderwood S. K. Murshid A. Prince T. (2009). The shock of aging: molecular chaperones and the heat shock response in longevity and aging–a mini-review. Gerontology 55 (5), 550–558. doi: 10.1159/000225957 19546513 PMC2754743

[B12] Castillejo C. de la Fuente J. I. Iannetta P. Botella M.Á. Valpuesta V. (2004). Pectin esterase gene family in strawberry fruit: study of FaPE1, a ripening-specific isoform. J. Exp. Bot. 55 (398), 909–918. doi: 10.1093/jxb/erh102 15020638

[B13] Chaudhary N. Nijhawan A. Khurana J. P. Khurana P. (2010). Carotenoid biosynthesis genes in rice: Structural analysis, genome-wide expression profiling and phylogenetic analysis. Mol. Genet. Genomics 283 (1), 13–33. doi: 10.1007/S00438-009-0495-X 19890663

[B14] Cheng G. Wang M. Zhang L. Wei H. Wang H. Lu J.. (2022). Overexpression of a cotton aquaporin gene GhTIP1;1-like confers cold tolerance in transgenic arabidopsis. Int. J. Mol. Sci. 23 (3), 1361. doi: 10.3390/ijms23031361 35163287 PMC8836057

[B15] Chomczynski P. Sacchi N. (1987). Single-step method of RNA isolation by acid guanidinium thiocyanate-phenol-chloroform extraction. Analytical Biochem. 162 (1), 156–159. doi: 10.1006/abio.1987.9999 2440339

[B16] Cui Y. Lu S. Li Z. Cheng J. Hu P. Zhu T.. (2020). CYCLIC NUCLEOTIDE-GATED ION CHANNELs 14 and 16 promote tolerance to heat and chilling in rice. Plant Physiol. 183 (4), 1794–1808. doi: 10.1104/PP.20.00591 32527735 PMC7401114

[B17] Cui Y. Peng Y. Zhang Q. Xia S. Ruan B. Xu Q.. (2021). Disruption of EARLY LESION LEAF 1, encoding a cytochrome P450 monooxygenase, induces ROS accumulation and cell death in rice. Plant J. 105 (4), 942–956. doi: 10.1111/TPJ.15079 33190327

[B18] Dong S. Sang L. Xie H. Chai M. Wang Z. Y. (2021). Comparative transcriptome analysis of salt stress-induced leaf senescence in medicago truncatula. Front. Plant Sci. 12. doi: 10.3389/FPLS.2021.666660 PMC8299074 34305965

[B19] Durian G. Sedaghatmehr M. Matallana-Ramirez L. P. Schilling S. M. Schaepe S. Guerra T.. (2020). Calcium-dependent protein kinase CPK1 controls cell death by *In vivo* phosphorylation of senescence master regulator ORE1. Plant Cell 32 (5), 1610–1625. doi: 10.1105/TPC.19.00810 32111670 PMC7203915

[B20] Du Z. Su Q. Wu Z. Huang Z. Bao J. Li J.. (2021). Genome-wide characterization of MATE gene family and expression profiles in response to abiotic stresses in rice (Oryza sativa). BMC Ecol. Evol. 21 (1), 1–14. doi: 10.1186/S12862-021-01873-Y 34243710 PMC8268253

[B21] Ellis C. M. Nagpal P. Young J. C. Hagen G. Guilfoyle T. J. Reed J. W. (2005). AUXIN RESPONSE FACTOR1 and AUXIN RESPONSE FACTOR2 regulate senescence and floral organ abscission in arabidopsis thaliana. Dev. (Cambridge England) 132 (20), 4563–4574. doi: 10.1242/DEV.02012 16176952

[B22] Fang Y. Xie K. Xiong L. (2014). Conserved miR164-targeted NAC genes negatively regulate drought resistance in rice. J. Exp. Bot. 65 (8), 2119–2135. doi: 10.1093/JXB/ERU072 24604734 PMC3991743

[B23] Fedina E. Yarin A. Mukhitova F. Blufard A. Chechetkin I. (2016). Brassinosteroid-induced changes of lipid composition in leaves of pisum sativum l. during senescence. Steroids 117, 25–28. doi: 10.1016/j.steroids.2016.10.009 27815033

[B24] Fröhlich M. Kutschera U. (1995). Changes in soluble sugars and proteins during development of rye coleoptiles. J. Plant Physiol. 146 (1–2), 121–125. doi: 10.1016/S0176-1617(11)81977-2

[B25] Graaff E. Schwacke R. Schneider A. Desimone M. Flügge U.-I. Kunze R. (2006). Transcription analysis of arabidopsis membrane transporters and hormone pathways during developmental and induced leaf senescence. Plant Physiol. 141 (2), 776–792. doi: 10.1104/pp.106.079293 16603661 PMC1475451

[B26] Gray W. M. (2004). Hormonal regulation of plant growth and development. PloS Biol. 2 (9), e311. doi: 10.1371/JOURNAL.PBIO.0020311 15367944 PMC516799

[B27] Gregersen P. L. Holm P. B. (2007). Transcriptome analysis of senescence in the flag leaf of wheat ( triticum aestivum l .). Plant Biotechnol. J. 5 (1), 192–206. doi: 10.1111/j.1467-7652.2006.00232.x 17207268

[B28] Guo Y. Cai Z. Gan S. (2004). Transcriptome of arabidopsis leaf senescence. Plant Cell Environ. 27 (5), 521–549. doi: 10.1111/j.1365-3040.2003.01158.x

[B29] Guo Y. Gan S.-S. (2012). Convergence and divergence in gene expression profiles induced by leaf senescence and 27 senescence-promoting hormonal, pathological and environmental stress treatments. Plant Cell Environ. 35 (3), 644–655. doi: 10.1111/j.1365-3040.2011.02442.x 21988545

[B30] Guo Y. Ren G. Zhang K. Li Z. Miao Y. Guo H. (2021). Leaf senescence: progression, regulation, and application. Mol. Horticulture 1, 1. doi: 10.1186/S43897-021-00006-9 PMC10509828 37789484

[B31] Häffner E. Konietzki S. Diederichsen E. (2015). Keeping control: The role of senescence and development in plant pathogenesis and defense. Plants 4 (3), 449–488. doi: 10.3390/PLANTS4030449 27135337 PMC4844401

[B32] Hao C. Yang Y. Du J. Deng X. W. Li L. (2022). The PCY-SAG14 phytocyanin module regulated by PIFs and miR408 promotes dark-induced leaf senescence in arabidopsis. Proc. Natl. Acad. Sci. U.S.A. 119 (3), 1–10. doi: 10.1073/pnas.2116623119 PMC8784109 35022242

[B33] Hinckley W. E. Brusslan J. A. (2020). Gene expression changes occurring at bolting time are associated with leaf senescence in arabidopsis. Plant Direct 4 (11), e00279. doi: 10.1002/pld3.279 33204935 PMC7649007

[B34] Houben M. Van de Poel B. (2019). 1-aminocyclopropane-1-carboxylic acid oxidase (ACO): The enzyme that makes the plant hormone ethylene. Front. Plant Sci. 10. doi: 10.3389/FPLS.2019.00695 PMC6549523 31191592

[B35] Huang Y. Guo Y. Liu Y. Zhang F. Wang Z. Wang H.. (2018). 9-cis-epoxycarotenoid dioxygenase 3 regulates plant growth and enhances multi-abiotic stress tolerance in rice. Front. Plant Sci. 9. doi: 10.3389/FPLS.2018.00162/BIBTEX PMC5845534 29559982

[B36] Huang Y. Jiao Y. Xie N. Guo Y. Zhang F. Xiang Z.. (2019). OsNCED5, a 9-cis-epoxycarotenoid dioxygenase gene, regulates salt and water stress tolerance and leaf senescence in rice. Plant Sci. 287, 110188. doi: 10.1016/J.PLANTSCI.2019.110188 31481229

[B37] Huo X. Wang C. Teng Y. Liu X. (2015). Identification of miRNAs associated with dark-induced senescence in arabidopsis. BMC Plant Biol. 15 (1), 1–12. doi: 10.1186/s12870-015-0656-5 26530097 PMC4632659

[B38] Hutin C. Nussaume L. Moise N. Moya I. Kloppstech K. Havaux M. (2003). Early light-induced proteins protect arabidopsis from photooxidative stress. Proc. Natl. Acad. Sci. U.S.A. 100 (8), 4921–4926. doi: 10.1073/PNAS.0736939100 12676998 PMC153656

[B39] Inada N. Sakai A. Kuroiwa H. Kuroiwa T. (1998). Three-dimensional analysis of the senescence program in rice (Oryza sativa l.) coleoptiles: Investigations by fluorescence microscopy and electron microscopy. Planta 206 (4), 585–597. doi: 10.1007/s004250050307 9637068

[B40] Inada N. Sakai A. Kuroiwa H. Kuroiwa T. (2002). “Three-dimensional progression of programmed death in the rice coleoptile,” in International review of cytology, vol. 218). (USA: Elsevier Masson SAS). doi: 10.1016/S0074-7696(02)18014-4 12199518

[B41] Jia M. Liu X. Xue H. Wu Y. Shi L. Wang R.. (2019). Noncanonical ATG8-ABS3 interaction controls senescence in plants. Nat. Plants 5 (2), 212–224. doi: 10.1038/S41477-018-0348-X 30664732 PMC6368864

[B42] Jiang Y. Liang G. Yang S. Yu D. (2014). Arabidopsis WRKY57 functions as a node of convergence for jasmonic acid – and auxin-mediated signaling in jasmonic acid-Induced leaf senescence. The Plant Cell 26 (1), 230–245. doi: 10.1105/tpc.113.117838 24424094 PMC3963572

[B43] Jung J. H. Lee H. J. Ryu J. Y. Park C. M. (2016). SPL3/4/5 integrate developmental aging and photoperiodic signals into the FT-FD module in arabidopsis flowering. Mol. Plant 9 (12), 1647–1659. doi: 10.1016/J.MOLP.2016.10.014 27815142

[B44] Kawai M. Uchimiya H. (2000). Coleoptile senescence in rice (Oryza sativa l.). Ann. Bot. 86 (2), 405–414. doi: 10.1006/anbo.2000.1199

[B45] Khan M. Rozhon W. Poppenberger B. (2014). The role of hormones in the aging of plants - a mini-review. Gerontology 60 (1), 49–55. doi: 10.1159/000354334 24135638

[B46] Kim J. H. Chung K. M. Woo H. R. (2011). Three positive regulators of leaf senescence in arabidopsis, ORE1, ORE3 and ORE9, play roles in crosstalk among multiple hormone-mediated senescence pathways. Genes Genomics 33, 373–381. doi: 10.1007/s13258-011-0044-y

[B47] Kim J. Kim J. H. Lyu J. Woo H. R. Lim P. O. (2018). New insights into the regulation of leaf senescence in arabidopsis. J. Exp. Bot. 69 (4), 787–799. doi: 10.1093/JXB/ERX287 28992051

[B48] Kim J. H. Woo H. R. Kim J. Lim P. O. Lee I. C. Choi S. H.. (2009). Trifurcate feed-forward regulation of age-dependent cell death involving miR164 in arabidopsis. Science 323 (5917), 1053–1057. doi: 10.1126/science.1166386 19229035

[B49] Kozomara A. Griffiths-Jones S. (2014). miRBase: annotating high confidence microRNAs using deep sequencing data. Nucleic Acids Res. 42 (D1), D68–D73. doi: 10.1093/NAR/GKT1181 24275495 PMC3965103

[B50] Kusaba M. Ito H. Morita R. Iida S. Sato Y. Fujimoto M.. (2007). Rice NON-YELLOW COLORING1 is involved in light-harvesting complex II and grana degradation during leaf senescence. Plant Cell 19 (4), 1362–1375. doi: 10.1105/tpc.106.042911 17416733 PMC1913755

[B51] Lee S. Jeong H. Lee S. Lee J. Kim S. Park J.. (2017). Molecular bases for differential aging programs between flag and second leaves during grain-filling in rice. Sci. Rep. 7 (1), 1–16. doi: 10.1038/s41598-017-07035-9 28821707 PMC5562787

[B52] Liang C. Wang Y. Zhu Y. Tang J. Hu B. Liu L.. (2014). OsNAP connects abscisic acid and leaf senescence by fine-tuning abscisic acid biosynthesis and directly targeting senescence-associated genes in rice. Proc. Natl. Acad. Sci. U. S. A. 111 (27), 10013–10018. doi: 10.1073/pnas.1321568111 24951508 PMC4103337

[B53] Li S. Castillo-González C. Yu B. Zhang X. (2017). The functions of plant small RNAs in development and in stress responses. Plant J. 90 (4), 654–670. doi: 10.1111/TPJ.13444 27943457

[B54] Li L. He Y. Zhang Z. Shi Y. Zhang X. Xu X.. (2021). OsNAC109 regulates senescence, growth and development by altering the expression of senescence- and phytohormone-associated genes in rice. Plant Mol. Biol. 105 (6), 637–654. doi: 10.1007/S11103-021-01118-Y 33543390 PMC7985107

[B55] Lim P. O. Kim H. J. Gil Nam H. (2007). Leaf senescence. Annu. Rev. Plant Biol. 58 (1), 115–136. doi: 10.1146/annurev.arplant.57.032905.105316 17177638

[B56] Lim J.-H. Paek N.-C. (2015). Quantitative trait locus mapping and candidate gene analysis for functional stay-green trait in rice. Plant Breed. Biotechnol. 3 (2), 95–107. doi: 10.9787/pbb.2015.3.2.095

[B57] Lin M. Pang C. Fan S. Song M. Wei H. Yu S. (2015). Global analysis of the gossypium hirsutum l. transcriptome during leaf senescence by RNA-seq. BMC Plant Biol. 15 (1), 1–18. doi: 10.1186/S12870-015-0433-5 25849479 PMC4342795

[B58] Li Z. Peng J. Wen X. Guo H. (2013). ETHYLENE-INSENSITIVE3 is a senescence-associated gene that accelerates age-dependent leaf senescence by directly repressing miR164 transcription in arabidopsis. Plant Cell 25 (9), 3311. doi: 10.1105/TPC.113.113340 24064769 PMC3809534

[B59] Livak K. J. Schmittgen T. D. (2001). Analysis of relative gene expression data using real-time quantitative PCR and the 2(-delta delta C(T)) method. Methods (San Diego Calif.) 25 (4), 402–408. doi: 10.1006/METH.2001.1262 11846609

[B60] Li N. Zhao M. Liu T. Dong L. Cheng Q. Wu J.. (2017). A novel soybean dirigent gene GmDIR22 contributes to promotion of lignan biosynthesis and enhances resistance to phytophthora sojae. Front. Plant Sci. 8, 1–12. doi: 10.3389/fpls.2017.01185 28725237 PMC5495835

[B61] Luu D. T. Maurel C. (2005). Aquaporins in a challenging environment: molecular gears for adjusting plant water status. Plant Cell Environ. 28 (1), 85–96. doi: 10.1111/J.1365-3040.2004.01295.X

[B62] Mao C. Lu S. Lv B. Zhang B. Shen J. He J.. (2017). A rice NAC transcription factor promotes leaf senescence *via* ABA biosynthesis. Plant Physiol. 174 (3), 1747–1763. doi: 10.1104/pp.17.00542 28500268 PMC5490923

[B63] Ma S. Quist T. M. Ulanov A. Joly R. Bohnert H. J. (2004). Loss of TIP1;1 aquaporin in arabidopsis leads to cell and plant death. Plant Journal : For Cell Mol. Biol. 40 (6), 845–859. doi: 10.1111/J.1365-313X.2004.02265.X 15584951

[B64] Maurel C. Verdoucq L. Luu D. T. Santoni V. (2008). Plant aquaporins: membrane channels with multiple integrated functions. Annu. Rev. Plant Biol. 59, 595–624. doi: 10.1146/ANNUREV.ARPLANT.59.032607.092734 18444909

[B65] Meyers B. C. Axtell M. J. Bartel B. Bartel D. P. Baulcombe D. Bowman J. L.. (2008). Criteria for annotation of plant MicroRNAs. THE Plant Cell Online 20 (12), 3186–3190. doi: 10.1105/tpc.108.064311 PMC2630443 19074682

[B66] Mohapatra P. K. Patro L. Raval M. K. Ramaswamy N. K. Biswal U. C. Biswal B. (2010). Senescence-induced loss in photosynthesis enhances cell wall β-glucosidase activity. Physiologia Plantarum 138 (3), 346–355. doi: 10.1111/J.1399-3054.2009.01327.X 20028477

[B67] Moschen S. Bengoa Luoni S. Di Rienzo J. A. Caro M. del P. Tohge T.. (2016). Integrating transcriptomic and metabolomic analysis to understand natural leaf senescence in sunflower. Plant Biotechnol. J. 14 (2), 719–734. doi: 10.1111/PBI.12422 26132509 PMC11629808

[B68] Narsai R. Edwards J. M. Roberts T. H. Whelan J. Joss G. H. Atwell B. J. (2015). Mechanisms of growth and patterns of gene expression in oxygen-deprived rice coleoptiles. Plant J. 82 (1), 25–40. doi: 10.1111/TPJ.12786 25650041

[B69] Noh Y. S. Amasino R. M. (1999). Identification of a promoter region responsible for the senescence-specific expression of SAG12. Plant Mol. Biol. 41, 2. doi: 10.1023/A:1006342412688 10579486

[B70] Pandian B. A. Sathishraj R. Djanaguiraman M. Prasad P. V. V. Jugulam M. (2020). Role of cytochrome P450 enzymes in plant stress response. Antioxidants (Basel Switzerland) 9 (5), E454–E454. doi: 10.3390/ANTIOX9050454 PMC7278705 32466087

[B71] Pan L. Zhao H. Yu Q. Bai L. Dong L. (2017). miR397/laccase gene mediated network improves tolerance to fenoxaprop-p-ethyl in beckmannia syzigachne and oryza sativa. Front. Plant Sci. 8. doi: 10.3389/FPLS.2017.00879/BIBTEX PMC5440801 28588605

[B72] Park S.-Y. Yu J.-W. Park J.-S. Li J. Yoo S.-C. Lee N.-Y.. (2007). The senescence-induced staygreen protein regulates chlorophyll degradation. Plant Cell 19 (5), 1649–1664. doi: 10.1105/tpc.106.044891 17513504 PMC1913741

[B73] Patel R. K. Jain M. (2012). NGS QC toolkit: A toolkit for quality control of next generation sequencing data. PloS One 7 (2):e30619. doi: 10.1371/journal.pone.0030619 22312429 PMC3270013

[B74] Poovaiah B. W. Leopold A. C. (1973). Deferral of leaf senescence with calcium. Plant Physiol. 52 (3), 236. doi: 10.1104/PP.52.3.236 16658538 PMC366476

[B75] Pruzinská A. Tanner G. Aubry S. Anders I. Moser S. Müller T.. (2005). Chlorophyll breakdown in senescent arabidopsis leaves. characterization of chlorophyll catabolites and of chlorophyll catabolic enzymes involved in the degreening reaction. Plant Physiol. 139 (1), 52–63. doi: 10.1104/pp.105.065870 16113212 PMC1203357

[B76] Qin J. Ma X. Yi Z. Tang Z. Meng Y. (2016). A transcriptome-wide study on the microRNA- and the argonaute 1-enriched small RNA-mediated regulatory networks involved in plant leaf senescence. Plant Biol. 18 (2), 197–205. doi: 10.1111/PLB.12373 26206233

[B77] Quirino B. F. Normanly J. Amasino R. M. (1999). Diverse range of gene activity during arabidopsis thaliana leaf senescence includes pathogen-independent induction of defense-related genes. Plant Mol. Biol. 40, 2. doi: 10.1023/A:1006199932265 10412905

[B78] Ranocha P. Chabannes M. Chamayou S. Danoun S. Jauneau A. Boudet A. M.. (2002). Laccase down-regulation causes alterations in phenolic metabolism and cell wall structure in poplar. Plant Physiol. 129 (1), 145. doi: 10.1104/PP.010988 12011346 PMC155879

[B79] Sağlam-Çağ S. (2007). The effect of epibrassinolide on senescence in wheat leaves. Biotechnol. Biotechnol. Equip. 21 (1), 63–65. doi: 10.1080/13102818.2007.10817415

[B80] Sakuraba Y. Schelbert S. Park S. Y. Han S. H. Lee B. D. Andrès C. B.. (2012). STAY-GREEN and chlorophyll catabolic enzymes interact at light-harvesting complex II for chlorophyll detoxification during leaf senescence in arabidopsis. Plant Cell 24 (2), 507–518. doi: 10.1105/TPC.111.089474 22366162 PMC3315229

[B81] Sasi J. M. Kumar C. V. Mani B. Bhardwaj A. R. Agarwal M. Katiyar-Agarwal S. (2019). Identification and characterization of miRNAs during flag leaf senescence in rice by high-throughput sequencing. Plant Physiol. Rep. 24 (1), 1–14. doi: 10.1007/S40502-019-0436-6

[B82] Schaller A. (2004). A cut above the rest: The regulatory function of plant proteases. Planta 220 (2), 183–197. doi: 10.1007/s00425-004-1407-2 15517349

[B83] Schelbert S. Aubry S. Burla B. Agne B. Kessler F. Krupinska K.. (2009). Pheophytin pheophorbide hydrolase (pheophytinase) is involved in chlorophyll breakdown during leaf senescence in arabidopsis. Plant Cell 21 (3), 767–785. doi: 10.1105/tpc.108.064089 19304936 PMC2671698

[B84] Schippers J. H. M. Jing H. Hille J. Dijkwel P. P. (2007). “Developmental and hormonal control of leaf senescence,” in Senescence processes in plants (Oxford: Blackwell Publishing), 145–170.

[B85] Schommer C. Palatnik J. F. Aggarwal P. Chételat A. Cubas P. Farmer E. E.. (2008). Control of jasmonate biosynthesis and senescence by miR319 targets. PloS Biol. 6 (9), e230. doi: 10.1371/journal.pbio.0060230 18816164 PMC2553836

[B86] Serafini-Fracassini D. Del Duca S. Monti F. Poli F. Sacchetti G. Bregoli A. M.. (2002). Transglutaminase activity during senescence and programmed cell death in the corolla of tobacco (Nicotiana tabacum) flowers. Cell Death Differentiation 9, 309–321. doi: 10.1038/sj.cdd.4400954 11859413

[B87] Shimoda Y. Ito H. Tanaka A. (2016). Arabidopsis STAY-GREEN, mendel’s green cotyledon gene, encodes magnesium-dechelatase. Plant Cell 28 (9), 2147–2160. doi: 10.1105/TPC.16.00428 27604697 PMC5059807

[B88] Sillanpää M. Kontunen-Soppela S. Luomala E. M. Sutinen S. Kangasjärvi J. Häggman H.. (2005). Expression of senescence-associated genes in the leaves of silver birch (Betula pendula). Tree Physiol. 25 (9), 1161–1172. doi: 10.1093/TREEPHYS/25.9.1161 15996959

[B89] Simpson K. Fuentes P. Quiroz-Iturra L. F. Flores-Ortiz C. Contreras R. Handford M.. (2018). Unraveling the induction of phytoene synthase 2 expression by salt stress and abscisic acid in daucus carota. J. Exp. Bot. 69 (16), 4113–4126. doi: 10.1093/JXB/ERY207 29860511 PMC6054239

[B90] Staden J. (1995). Hormonal control of carnation flower senescence. Acta Hortic. 405, 232–239. doi: 10.17660/ACTAHORTIC.1995.405.30

[B91] Stocks M. B. Moxon S. Mapleson D. Woolfenden H. C. Mohorianu I. Folkes L.. (2012). The UEA sRNA workbench: a suite of tools for analysing and visualizing next generation sequencing microRNA and small RNA datasets. Bioinf. (Oxford England) 28 (15), 2059–2061. doi: 10.1093/bioinformatics/bts311 PMC3400958 22628521

[B92] Swida-Barteczka A. Szweykowska-Kulinska Z. (2019). Micromanagement of developmental and stress-induced senescence: The emerging role of MicroRNAs. Genes 10 (3), 210. doi: 10.3390/GENES10030210 30871088 PMC6470504

[B93] Szklarczyk D. Gable A. L. Nastou K. C. Lyon D. Kirsch R. Pyysalo S.. (2021). The STRING database in 2021: customizable protein-protein networks, and functional characterization of user-uploaded gene/measurement sets. Nucleic Acids Res. 49 (D1), 605–612. doi: 10.1093/nar/gkaa1074 PMC7779004 33237311

[B94] Takasaki H. Maruyama K. Takahashi F. Fujita M. Yoshida T. Nakashima K.. (2015). SNAC-as, stress-responsive NAC transcription factors, mediate ABA-inducible leaf senescence. Plant journal: Cell Mol. Biol. 84 (6), 1114–1123. doi: 10.1111/tpj.13067 26518251

[B95] Tamás L. Čiamporová M. Luxová M. (1998). Accumulation of pathogenesis-related proteins in barley leaf intercellular spaces during leaf senescence. Biol. Plantarum 41, 3. doi: 10.1023/A:1001814930794

[B96] Tanabe S. (2005). A novel cytochrome P450 is implicated in brassinosteroid biosynthesis *via* the characterization of a rice dwarf mutant, dwarf11, with reduced seed length. Plant Cell Online 17, 776–790. doi: 10.1105/tpc.104.024950 PMC1069698 15705958

[B97] Tang Y. Li M. Chen Y. Wu P. Wu G. Jiang H. (2011). Knockdown of OsPAO and OsRCCR1 cause different plant death phenotypes in rice. J. Plant Physiol. 168 (16), 1952–1959. doi: 10.1016/J.JPLPH.2011.05.026 21807436

[B98] Taylor R. C. Dillin A. (2011). Aging as an event of proteostasis collapse. Cold Spring Harbor Perspect. Biol. 3 (5), 1–17. doi: 10.1101/CSHPERSPECT.A004440 PMC3101847 21441594

[B99] Thatcher S. R. Burd S. Wright C. Lers A. Green P. J. (2015). Differential expression of miRNAs and their target genes in senescing leaves and siliques: insights from deep sequencing of small RNAs and cleaved target RNAs. Plant Cell Environ. 38 (1), 188–200. doi: 10.1111/pce.12393 24965556 PMC4304344

[B100] Thimm O. BlaÈ sing O. Gibon Y. Nagel A. Meyer S. KruÈ ger P.. (2004). MAPMAN: A user-driven tool to display genomics data sets onto diagrams of metabolic. Plant J. 37, 914–939. doi: 10.1111/j.1365-313x.2004.02016.x 14996223

[B101] Trapnell C. Pachter L. Salzberg S. L. (2009). TopHat: Discovering splice junctions with RNA-seq. Bioinformatics 25 (9), 1105–1111. doi: 10.1093/bioinformatics/btp120 19289445 PMC2672628

[B102] Trivedi R. Jurivich D. A. (2020). A molecular perspective on age-dependent changes to the heat shock axis. Exp. Geronto. 137, 110969. doi: 10.1016/J.EXGER.2020.110969 32407864

[B103] Tsuzuki M. Futagami K. Shimamura M. Inoue C. Kunimoto K. Oogami T.. (2019). An early arising role of the MicroRNA156/529-SPL module in reproductive development revealed by the liverwort marchantia polymorpha. Curr. Biology : CB 29 (19), 3307–3314.e5. doi: 10.1016/J.CUB.2019.07.084 31543452

[B104] Upadhyay N. Kar D. Deepak Mahajan B. Nanda S. Rahiman R. Panchakshari N.. (2019). The multitasking abilities of MATE transporters in plants. J. Exp. Bot. 70 (18), 4643–4656. doi: 10.1093/JXB/ERZ246 31106838

[B105] Wang Z. Qian C. Guo X. Liu E. Mao K. Mu C.. (2016). ELS1, a novel MATE transporter related to leaf senescence and iron homeostasis in arabidopsis thaliana. Biochem. Biophys. Res. Commun. 476 (4), 319–325. doi: 10.1016/J.BBRC.2016.05.121 27233612

[B106] Wan F. Zhang L. Tan M. Wang X. Wang G. L. Qi M.. (2022). Genome-wide identification and characterization of laccase family members in eggplant (Solanum melongena l.). PeerJ 10, 1–24. doi: 10.7717/peerj.12922 PMC8868016 35223206

[B107] Wei K. Chen H. (2018). Global identification, structural analysis and expression characterization of cytochrome P450 monooxygenase superfamily in rice. BMC Genomics 19 (1), 1–18. doi: 10.1186/s12864-017-4425-8 29320982 PMC5764023

[B108] Woo H. R. Chung K. M. Park J.-H. Oh S. A. Ahn T. Hong S. H.. (2001). ORE9, an f-box protein that regulates leaf senescence in arabidopsis. Plant Cell 13 (8), 1779. doi: 10.1105/TPC.010061 11487692 PMC139127

[B109] Wu A. Allu A. D. Garapati P. Siddiqui H. Dortay H. Zanor M. I.. (2012). JUNGBRUNNEN1, a reactive oxygen species–responsive NAC transcription factor, regulates longevity in arabidopsis. Plant Cell 24 (2), 482–506. doi: 10.1105/TPC.111.090894 22345491 PMC3315228

[B110] Wu X. Ding D. Shi C. Xue Y. Zhang Z. Tang G.. (2016). microRNA-dependent gene regulatory networks in maize leaf senescence. BMC Plant Biol. 16, 73. doi: 10.1186/s12870-016-0755-y 27000050 PMC4802599

[B111] Xin M. Wang Y. Yao Y. Xie C. Peng H. Ni Z.. (2010). Diverse set of microRNAs are responsive to powdery mildew infection and heat stress in wheat (Triticum aestivum l.). BMC Plant Biol. 10, 123. doi: 10.1186/1471-2229-10-123 20573268 PMC3095282

[B112] Xu X. Bai H. Liu C. Chen E. Chen Q. Zhuang J.. (2014). Genome-wide analysis of microRNAs and their target genes related to leaf senescence of rice. PloS One 9 (12), e114313. doi: 10.1371/journal.pone.0114313 25479006 PMC4257594

[B113] Yang S. D. Seo P. J. Yoon H. K. Park C. M. (2011). The arabidopsis NAC transcription factor VNI2 integrates abscisic acid signals into leaf senescence *via* the COR/RD genes. Plant Cell 23 (6), 2155–2168. doi: 10.1105/tpc.111.084913 21673078 PMC3160032

[B114] Yoshida S. Forno D. A. Cock J. H. Gomez K. A. (1976). Laboratory manual for physiological studies of rice (Manila: International Rice Research Institute;), 61–66.

[B115] Yuan S. Li Z. Li D. Yuan N. Hu Q. Luo H. (2015). Constitutive expression of rice MicroRNA528 alters plant development and enhances tolerance to salinity stress and nitrogen starvation in creeping bentgrass. Plant Physiol. 169 (1), 576. doi: 10.1104/PP.15.00899 26224802 PMC4577425

[B116] Zentgraf U. Andrade-Galan A. G. Bieker S. (2022). Specificity of H2O2 signaling in leaf senescence: is the ratio of H2O2 contents in different cellular compartments sensed in arabidopsis plants? Cell. Mol. Biol. Lett. 27 (1), 1–19. doi: 10.1186/S11658-021-00300-W 34991444 PMC8903538

[B117] Zhang X. Ju H. W. Chung M. S. Huang P. Ahn S. J. Kim C. S. (2011). The r-r-type MYB-like transcription factor, AtMYBL, is involved in promoting leaf senescence and modulates an abiotic stress response in arabidopsis. Plant Cell Physiol. 52 (1), 138–148. doi: 10.1093/pcp/pcq180 21097474

[B118] Zhang W. Y. Xu Y. C. Li W. L. Yang L. Yue X. Zhang X. S.. (2014). Transcriptional analyses of natural leaf senescence in maize. PloS One 9 (12), e115617. doi: 10.1371/journal.pone.0115617 25532107 PMC4274115

[B119] Zhu H. Chen C. Zeng J. Yun Z. Liu Y. Qu H.. (2020). MicroRNA528, a hub regulator modulating ROS homeostasis ** *via* ** targeting of a diverse set of genes encoding copper-containing proteins in monocots. New Phytol. 225 (1), 385–399. doi: 10.1111/NPH.16130 31429090

