# Corrigendum: Integrated transcriptomics and miRNAomics provide insights into the complex multi-tiered regulatory networks associated with coleoptile senescence in rice

**DOI:** 10.3389/fpls.2023.1163471

**Published:** 2023-02-27

**Authors:** Jyothish Madambikattil Sasi, Cheeni VijayaKumar, Bharti Kukreja, Roli Budhwar, Rohit Nandan Shukla, Manu Agarwal, Surekha Katiyar-Agarwal

**Affiliations:** ^1^Department of Plant Molecular Biology, University of Delhi South Campus, New Delhi, India; ^2^Department of Botany, University of Delhi, Delhi, India; ^3^Bionivid Technology Pvt. Limited, Bengaluru, Karnataka, India

**Keywords:** rice, coleoptile, senescence, transcriptome, miRNAs, regulation

In the published article, there was an error in the **Funding** statement. The **Funding** statement previously said:

“The financial support from University Grants Commission (sanction order # 41-512/2012), Government of India; Department of Biotechnology, Government of India and Faculty Research Programme (Institute of Eminence), University of Delhi (sanction order # IOE/FRP/LS/2020/27) are acknowledged. JMS and CV are thankful to UGC for Basic Science Research (BSR) fellowships.”.

The corrected **Funding** statement appears below:

The financial support from University Grants Commission (sanction order # 41-512/2012), Government of India; Department of Biotechnology, Government of India and Faculty Research Programme (Institute of Eminence), University of Delhi (sanction orders # IoE/FRP/2020/27 and IoE/2021/12/FRP) are acknowledged. JMS and CV are thankful to UGC for Basic Science Research (BSR) fellowships.

The authors apologize for this error and state that this does not change the scientific conclusions of the article in any way. The original article has been updated.

