# Supplementary material for: Integrated transcriptomics and miRNAomics provide insights into the complex multi-tiered regulatory networks associated with coleoptile senescence in rice
Source: Front Plant Sci. 2022 Oct 12;13:985402. doi: 10.3389/fpls.2022.985402 (PMC9597502; doi:10.3389/fpls.2022.985402)
Supplement: Supplementary file 1 [file Presentation_1.pptx]

## Slide 1
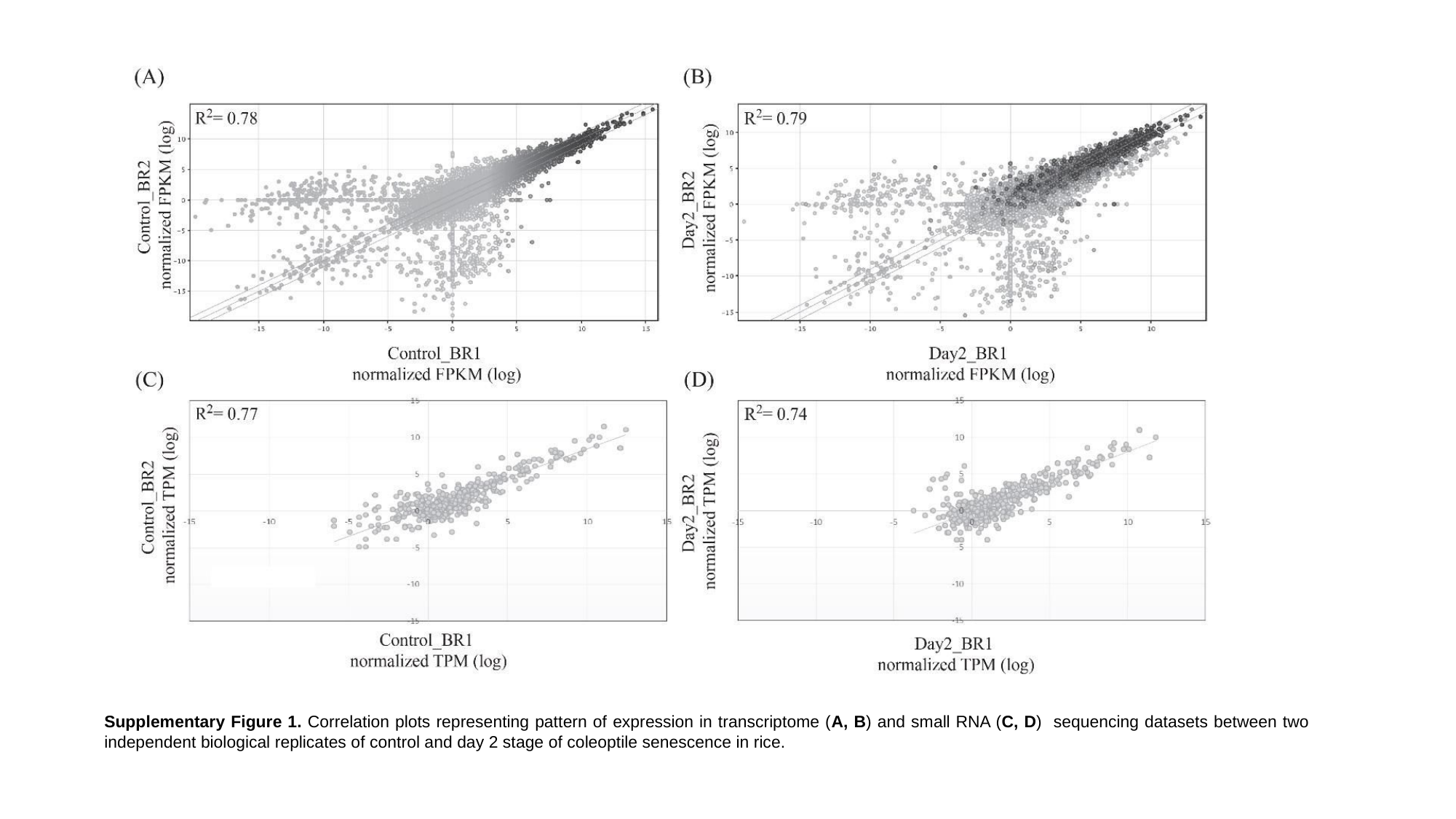

Supplementary Figure 1. Correlation plots representing pattern of expression in transcriptome (A, B) and small RNA (C, D) sequencing datasets between two independent biological replicates of control and day 2 stage of coleoptile senescence in rice.

## Slide 2
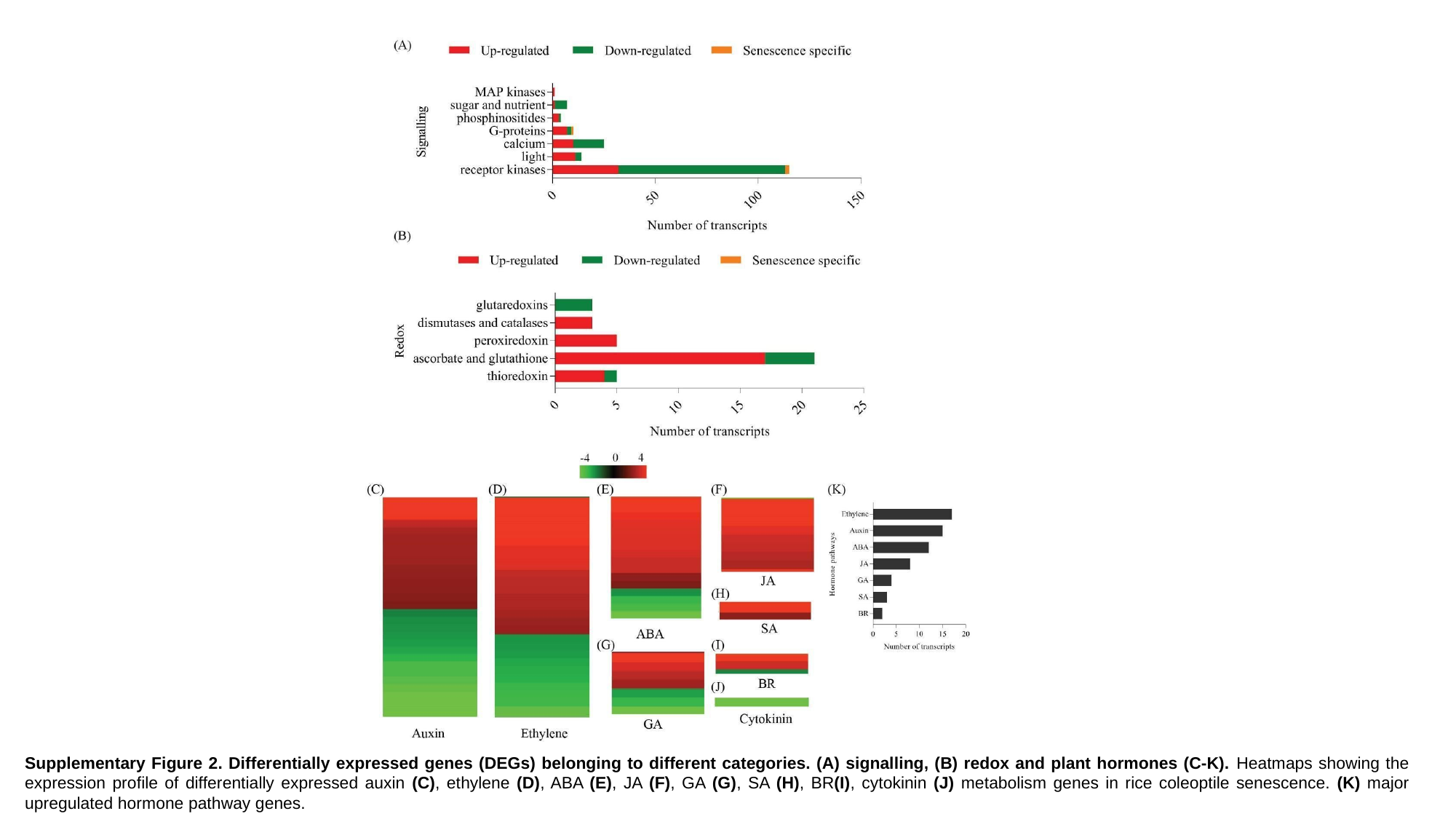

Supplementary Figure 2. Differentially expressed genes (DEGs) belonging to different categories. (A) signalling, (B) redox and plant hormones (C-K). Heatmaps showing the expression profile of differentially expressed auxin (C), ethylene (D), ABA (E), JA (F), GA (G), SA (H), BR(I), cytokinin (J) metabolism genes in rice coleoptile senescence. (K) major upregulated hormone pathway genes.

## Slide 3
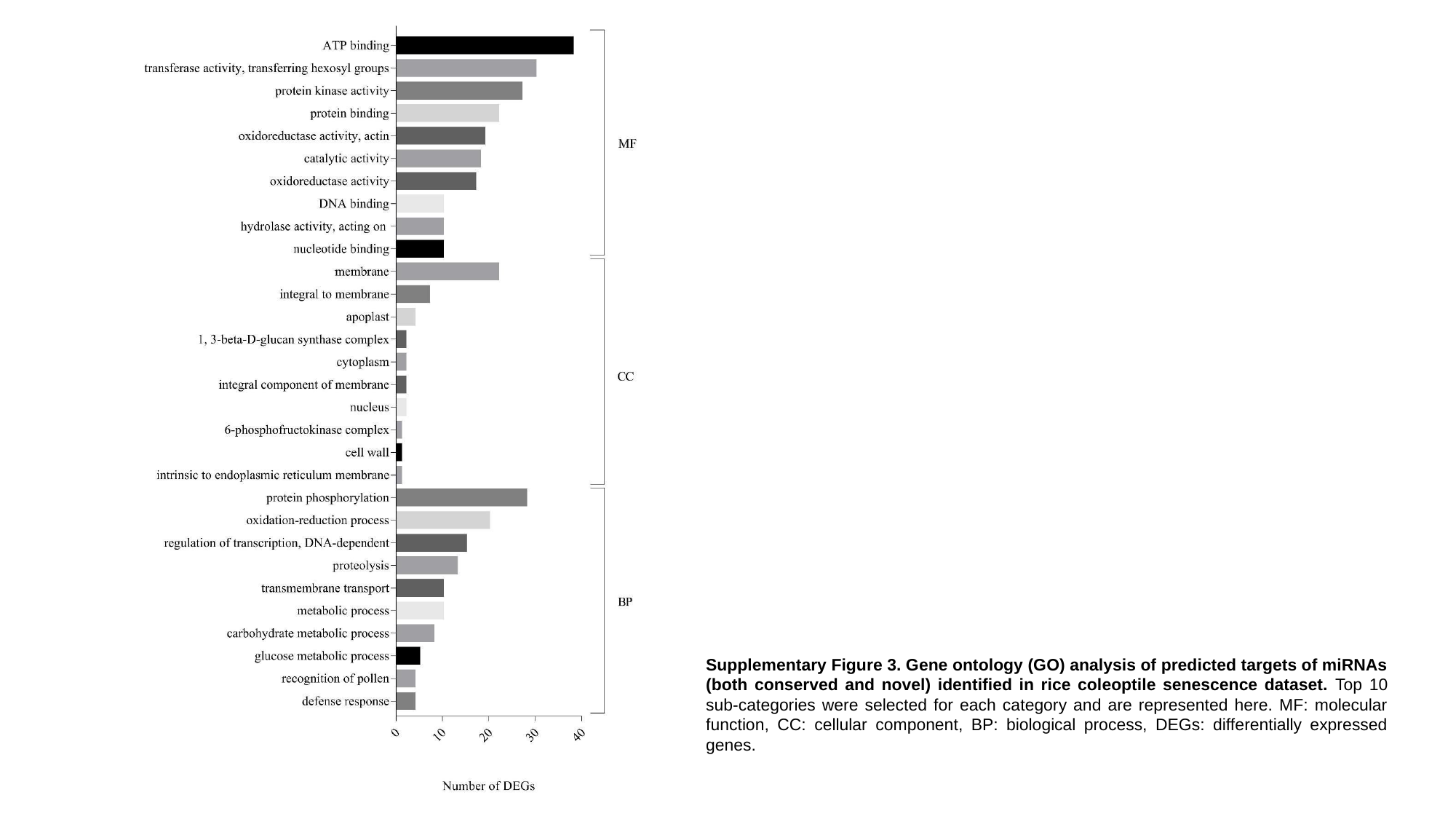

Supplementary Figure 3. Gene ontology (GO) analysis of predicted targets of miRNAs (both conserved and novel) identified in rice coleoptile senescence dataset. Top 10 sub-categories were selected for each category and are represented here. MF: molecular function, CC: cellular component, BP: biological process, DEGs: differentially expressed genes.
