# Supplementary material for: Integrated transcriptomics and miRNAomics provide insights into the complex multi-tiered regulatory networks associated with coleoptile senescence in rice
Source: Front Plant Sci. 2022 Oct 12;13:985402. doi: 10.3389/fpls.2022.985402 (PMC9597502; doi:10.3389/fpls.2022.985402)
Supplement: Supplementary file 2 [file Table_1.docx]

**Supplementary Table 1. Quality assessment of raw data obtained by sequencing of RNA-seq libraries.** Raw reads were subjected to quality assessment by employing NGS-QC tool kit. The total number of raw reads and high quality reads are indicated for individual libraries.

| S. No. | Library Name | Total No. of Reads  (million) | High Quality Reads  (million) | Low Quality Reads  (million) |
| --- | --- | --- | --- | --- |
| 1. | Control_BR1 | 28.67 | 28.04 | 0.63 |
| 2. | Day 2_BR1 | 29.49 | 28.81 | 0.67 |
| 3. | Control_BR2 | 36.55 | 35.54 | 1.01 |
| 4. | Day 2_BR2 | 28.07 | 27.43 | 0.64 |
