# Supplementary material for: Integrated transcriptomics and miRNAomics provide insights into the complex multi-tiered regulatory networks associated with coleoptile senescence in rice
Source: Front Plant Sci. 2022 Oct 12;13:985402. doi: 10.3389/fpls.2022.985402 (PMC9597502; doi:10.3389/fpls.2022.985402)
Supplement: Supplementary file 3 [file Table_2.docx]

**Supplementary Table 2.** **Summary of data filtration of small RNA sequencing data of rice coleoptile senescence.** Total number of redundant and unique sequencing reads obtained at each step of elimination pipeline are presented.

| Category | Number of redundant reads | Number of unique reads |
| --- | --- | --- |
| Total sequence reads | 393599652 | 84713047 |
| Sequences after adapter removal | 383611971 | 70607583 |
| Sequence length (<16 & >30 nt) | 289826024 | 60651256 |
| Low complexity sequences | 80624 | 12906 |
| Invalid sequences | 118519 | 67131 |
| rRNA/tRNA matches | 84242274 | 430258 |
| Putative small RNA population |  | 205384607 |
